# Exogenous Classic Phytohormones Have Limited Regulatory Effects on Fructan and Primary Carbohydrate Metabolism in Perennial Ryegrass (*Lolium perenne* L.)

**DOI:** 10.3389/fpls.2015.01251

**Published:** 2016-01-20

**Authors:** Anna Gasperl, Annette Morvan-Bertrand, Marie-Pascale Prud'homme, Eric van der Graaff, Thomas Roitsch

**Affiliations:** ^1^Institute of Plant Sciences, Karl-Franzens-Universität GrazGraz, Austria; ^2^Normandie UniversitéCaen, France; ^3^UMR 950 Ecophysiologie Végétale, Agronomie et Nutritions NCS, Université de Caen NormandieCaen, France; ^4^INRA, UMR 950 Ecophysiologie Végétale, Agronomie et Nutritions NCSCaen, France

**Keywords:** enzymatic activity, fructan exohydrolase, fructan metabolism, fructosyltransferase, perennial ryegrass, phytohormones, primary carbohydrate metabolism

## Abstract

Fructans are polymers of fructose and one of the main constituents of water-soluble carbohydrates in forage grasses and cereal crops of temperate climates. Fructans are involved in cold and drought resistance, regrowth following defoliation and early spring growth, seed filling, have beneficial effects on human health and are used for industrial processes. Perennial ryegrass (*Lolium perenne* L.) serves as model species to study fructan metabolism. Fructan metabolism is under the control of both synthesis by fructosyltransferases (FTs) and breakdown through fructan exohydrolases (FEHs). The accumulation of fructans can be triggered by high sucrose levels and abiotic stress conditions such as drought and cold stress. However, detailed studies on the mechanisms involved in the regulation of fructan metabolism are scarce. Since different phytohormones, especially abscisic acid (ABA), are known to play an important role in abiotic stress responses, the possible short term regulation of the enzymes involved in fructan metabolism by the five classical phytohormones was investigated. Therefore, the activities of enzymes involved in fructan synthesis and breakdown, the expression levels for the corresponding genes and levels for water-soluble carbohydrates were determined following pulse treatments with ABA, auxin (AUX), ethylene (ET), gibberellic acid (GA), or kinetin (KIN). The most pronounced fast effects were a transient increase of FT activities by AUX, KIN, ABA, and ET, while minor effects were evident for 1-FEH activity with an increased activity in response to KIN and a decrease by GA. Fructan and sucrose levels were not affected. This observed discrepancy demonstrates the importance of determining enzyme activities to obtain insight into the physiological traits and ultimately the plant phenotype. The comparative analyses of activities for seven key enzymes of primary carbohydrate metabolism revealed no co-regulation between enzymes of the fructan and sucrose pool.

## Introduction

Fructans are β(2,1) and/or β(2,6) linked polymers of fructose. Fructans are one of the main constituents of water-soluble carbohydrates (WSC) in forage grasses and cereal crops of temperate climates (Hendry, 1993) and are involved in cold and drought resistance (Livingston et al., 2009), grain filling (Xue et al., 2008; Zhang et al., 2015) and regrowth following defoliation and early spring growth (Morvan-Bertrand et al., 2001a; Prud'homme et al., 2007). In addition, fructans are under increasing interest as functional food supplement because of their beneficial effects on human health. Fructans are low caloric sweeteners, fat substitutes and important immunomodulators (Vijn and Smeekens, 1999; Ritsema and Smeekens, 2003; Vogt et al., 2013; Apolinário et al., 2014; Peshev and Van den Ende, 2014). Fructan accumulating crops have also attracted interest as carbohydrate source for the industrial conversion of biomass to chemicals, materials and bioenergy. The key primary degradation compound from the carbohydrate fraction is 5-hydroxymethylfurfural (Gallezot, 2012), which serves as one of the most promising platform molecules for future sustainable production of an unequaled wide range of bio-based products including esters, biopolymers, pharmaceuticals, food ingredients, agrochemicals, and biofuel. Perennial ryegrass (*Lolium perenne* L.) serves as model species to study fructan metabolism, because it is the predominant forage grass in European agriculture and studied intensively at the genomic, physiological and biochemical level (Prud'homme et al., 2007; Lee et al., 2010). The main focus of breeding is on ryegrass varieties with increased WSC levels (Turner et al., 2006). These are sought to improve animal productivity due to improved nitrogen use efficiency. Attempts including conventional breeding or genetic modifications are hampered, because little is known about regulatory factors of fructan metabolism (Rasmussen et al., 2009).

Fructan pool size is the result of the balance between biosynthesis from sucrose by fructosyl transferases (FTs EC 2.4.1.99, 2.4.1.100) and degradation by fructan exohydrolases (FEHs EC 3.2.1.153). In perennial ryegrass, four FT activities are required to produce the complement of fructans from sucrose; sucrose:sucrose 1-fructosyltransferase (1-SST), fructan:fructan 1-fructosyltransferase (1-FFT), fructan:fructan 6Gfructosyltransferase (6G-FFT), and 6-sucrose:fructan fructosyltransferase (6-SFT) activities (Pavis et al., 2001). These four FT activities are carried by three proteins; a 1-SST (Chalmers et al., 2003), a 6G-FFT/1-FFT (Lasseur et al., 2006) and a 6-SFT (Lasseur et al., 2011). FEHs differ by the preferential linkage, β(2,1) and/or β(2,6) on which they act and a 1-FEH and a 6-FEH have been identified in perennial ryegrass (Lothier et al., 2007, 2014). The accumulation of fructans can be increased by high sucrose levels (Pollock et al., 2003; Valluru et al., 2008) and abiotic stress conditions (Kerepesi et al., 2004; Ruuska et al., 2008; Valluru et al., 2008). Fructan accumulation is accompanied by high FT activities and corresponding mRNA levels in sink tissues (Lasseur et al., 2006; Lothier et al., 2014).

**Figure 1:**
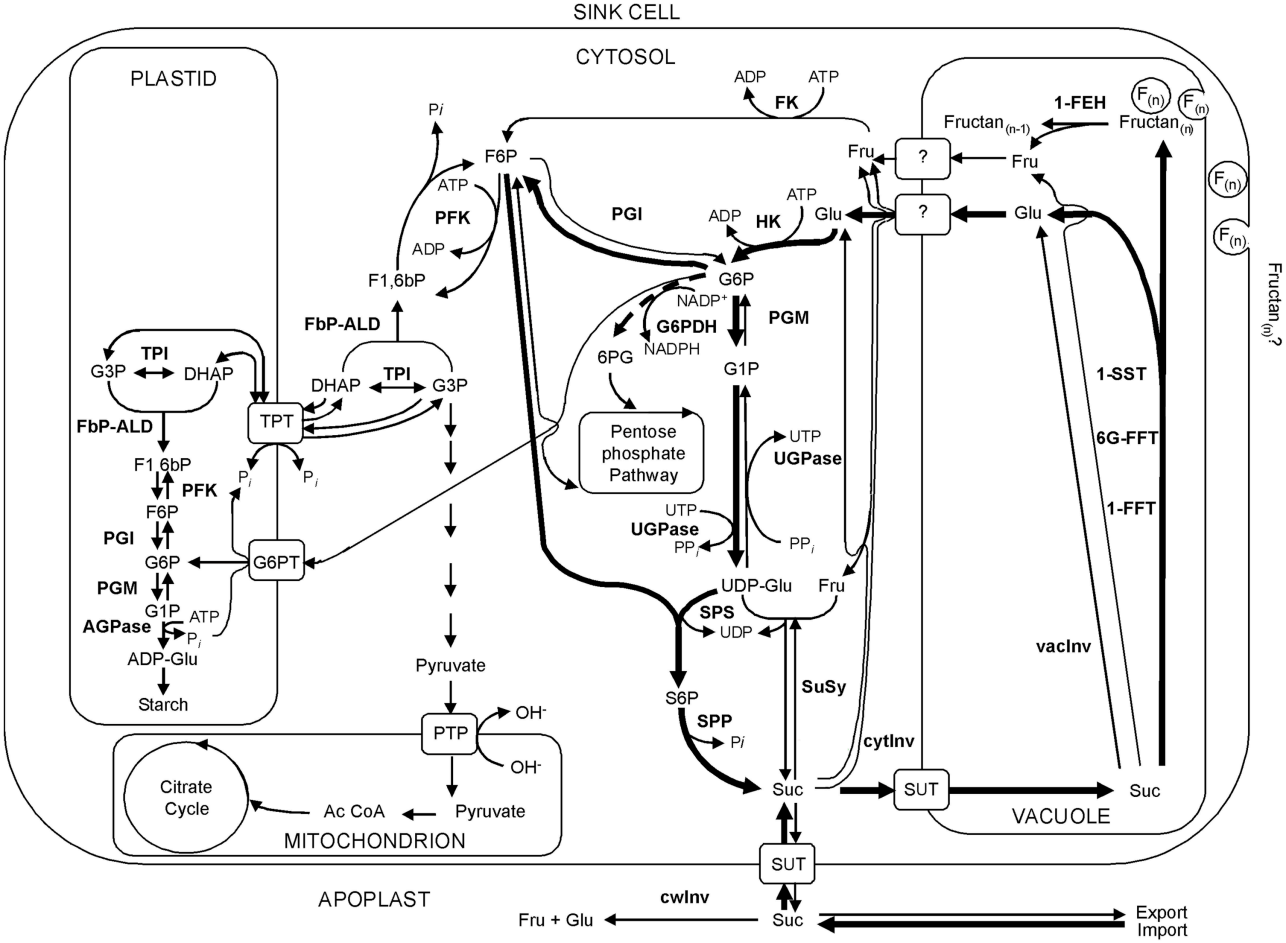
**Model for fructan and primary carbohydrate metabolism in sink cells of perennial ryegrass**. Bold arrows indicate preferential fluxes of carbon to induce fructan accumulation. The bold dotted arrow indicates the competition for G6P by G6PDH. Abbreviations: Ac Co A, acetyl coenzyme A; ADP, adenosine triphosphate; ADP-Gluc, adenosine triphosphate glucose; AGPase, adenosine diphosphate glucose pyrophosphorylase; ATP, adenosine triphosphate; DHAP, dihydroxyacetone phosphate; FbP-ALD, fructose-1,6-bisphosphate dependent aldolase; F1,6bP, fructose-1,6-bisphosphate; F6P, fructose-6-phosphate; FK, fructokinase; Fru, fructose; G1P, glucose-1-phosphate; G3P, 3-phospho glycerate; G6P, glucose-6-phosphate; G6PT, glucose-6-phosphat translocator; Glu, glucose; HK, hexokinase; 6PG, 6-phospho gluconate; NADP+, nicotinamid adenine dinucleotide phosphate(_ox,_); NADPH, nicotinamid adenine dinucleotide phosphate(_red,_); PFK, phosphofructokinase; PTP, pyruvate transport protein; SPP, sucrose phosphate phosphatase; SPS, sucrose phosphate synthase; Suc, sucrose; SuSy, sucrose synthase; SUT, sucrose transporter; TPI, triosephosphate isomerase; TPT, triose-phosphate/phosphate translocator; UDP, uracyl diphosphate; UDP-Glu, uridinyl diphosphate glucose; UTP, uridine triphosphate.

Considering the importance of the disaccharide sucrose as substrate for fructan biosynthesis (Van den Ende et al., 1996) and knowing that it is an inhibitor of fructan breakdown (Lothier et al., 2007, 2010), the availability of this molecule is also one of the main determinants for fructan production. In sink tissues, the supply of sucrose depends both on its import and metabolism (Figure 1, based on Offler and Patrick, 1999). In perennial ryegrass, the sucrose transporter LpSUT1 is supposed to play a key role in lateral partitioning of sucrose between the vascular tissue and the sites of fructan synthesis and degradation (Berthier et al., 2009, 2014). Besides transport, sucrose availability is greatly regulated by the activity of invertase enzymes (EC 3.2.1.26), cleaving sucrose in the hexoses glucose and fructose (Roitsch and González, 2004). A futile cycle of sucrose driven by invertases was found in source and sink tissue of perennial ryegrass (see Lattanzi et al., 2012 and references therein). Hexoses are likewise released during fructan accumulation (glucose) or degradation (fructose). Hence, futile cycling of sucrose might be crucial for the regulation of fructan metabolism. Sucrose is synthesized in the cytosol from fructose-6-phosphate via an enzyme cascade including phosphoglucoisomerase (PGI EC 5.3.1.9) for reversible conversion of fructose-6-phosphate (F6P) to glucose-6-phosphate (G6P), phosphoglucomutase (PGM EC 5.4.2.2), UDP-glucose pyrophosphorylase (UGPase EC 2.7.7.9), sucrose-phosphate synthase (SPS EC 2.4.1.14), and sucrose-phosphate phosphatase (SPP EC 3.1.3.24). Fructokinase (FK EC 2.7.1.4) and hexokinase (HK EC 2.7.1.1) provide hexose-phosphates. Induction of sucrose synthesis (SPS activity) is dependent on the availability of G6P (Halford et al., 2011). Therefore, the activities for these key enzymes involved in primary carbohydrate metabolism are additional factors that need to be addressed when studying fructan metabolism. Invertases cleave sucrose, but were also reported to degrade lowDP fructans, probably as side activity (Cairns, 1993, 2003) and thereby affect the sucrose pool available as substrate for fructan metabolism. Assuming a vacuolar localization of fructan metabolism (Darwen and John, 1989) and soluble acid invertase activity in the same organelle (Livingston et al., 2009), especially vacInv activity could be important in relation to fructan metabolism to compete for the same substrate. In this study, we focused on enzymes in primary carbohydrate metabolism that directly affect the sucrose pool via degradation: cell wall invertase (cwInv), cytosolic invertase (cytInv), and, vacuolar invertase (vacInv). In addition, the activities of PGI and PGM (glycolysis), glucose-6-phosphate dehydrogenase (G6PDH 1.1.1.49) (oxidative pentosephosphatecycle) and UGPase (resynthesis of sucrose) were determined. Because G6PDH competes for G6P, its activity is also of considerable importance. This is especially of interest, since expression and activity of a cytosolic G6PDH isoform was activated via sugars (glucose, fructose, sucrose) in potato leaf discs (Hauschild and von Schaewen, 2003).

Given the important role of phytohormones, especially that of abscisic acid (ABA) in the regulation of stress responses toward drought, salt and cold (Seki et al., 2002a,b; Shinozaki et al., 2003; Nakashima et al., 2009), the possible regulation of FT and FEH activities by phytohormones has to be considered to understand fructan metabolism. Only very few studies addressed the effect of phytohormones in the regulation of FT and FEH enzyme activities and/or corresponding gene expression in monocot species. ABA application was shown to increase both freezing tolerance and soluble sugar levels in barley (Bravo et al., 1998), and alter fructan levels (Kerepesi et al., 2004), fructan enzyme activities (Yang et al., 2004) and *FT* and *FEH* mRNA levels in wheat (Ruuska et al., 2008). However, these studies were performed on cereals during the generative stage and employed long exposures to the exogenous ABA. The role of ABA, kinetin (KIN), methyl jasmonate and salicylic acid was studied in agave, but this study was limited to *1-SST* and *1-FFT* expression using plants grown in liquid medium and long exposure (24–42 days) to these exogenous phytohormones (Suárez-González et al., 2014). So far, perennial ryegrass studies addressed only the role of gibberellins (GA) in fructan metabolism after defoliation (Morvan et al., 1997; Morvan-Bertrand et al., 2001b). The increase of FEH activity following defoliation was strongly inhibited by spraying of uniconazole, an inhibitor of the biosynthesis of growth regulators including GA, and this inhibition was reversed by a simultaneous treatment with GA_3_ (Morvan et al., 1997). These results suggested that the increase of FEH activity was mediated by a biosynthesis of GAs, but the level of GA_1_, considered as the most active GA, decreased simultaneously with the increase of FEH activity following defoliation (Morvan-Bertrand et al., 2001b). Altogether, the results indicate that GA_1_ is not positively involved in the control of FEH activity during regrowth and that the effect of uniconazole on FEH activity could have been mediated by changes in growth regulators other than GAs. Indeed, uniconazole is a strong inhibitor of ABA 8'-hydroxylase and can cause an increase in ABA content (Saito et al., 2006).

To assess the potential of phytohormones as triggers for short term changes in fructan and primary carbohydrate metabolism of perennial ryegrass, the set of the classical five phytohormones, ABA, ethylene (ET), auxin (AUX), KIN, and GA, suggested to play a role in stress related changes of primary carbohydrate (Roitsch, 1999; Roitsch and González, 2004; Cardi et al., 2011) and fructan metabolism (Morvan et al., 1997; Tase and Fuji, 1997; Bravo et al., 1998; Morvan-Bertrand et al., 2001b; Suárez-González et al., 2014; Valluru, 2015; Zhang et al., 2015), was tested. We assessed sugar levels, fructan enzyme activities and corresponding mRNA expression levels, sucrose transporter mRNA expression level and activities for key enzymes involved in primary carbohydrate metabolism up to 48 h following exogenously applied phytohormones via a single spraying (pulse) treatment. This exogenic application should provide insight on the fast regulatory roles these phytohormones play in fructan and primary carbohydrate metabolism in the basal first cm of the stubble, corresponding to the plant parts with highest fructan accumulation (mature leaf sheaths) and turnover (elongating leaf bases and immature leaf sheaths; Morvan-Bertrand et al., 2001a). After cold treatment, changes in low DP fructans and sucrose in perennial ryegrass were reported only after 4 days of cold treatment (Hisano et al., 2008). Considering the suggested role of phytohormones in stress responses and the fact that we directly applied phytohormones, we assessed physiological and gene expression changes within 2 days (24 and 48 h after phytohormone spraying) to minimize pleiotropic effects related to long exposure of non-physiological concentrations.

## Materials and methods

### Plant material

Perennial ryegrass (*L. perenne* L. genotype Aberchoice) was sown densely in vermiculite (Pull Rhenen, The Netherlands) in 6 cm pots and transferred into a growth cabinet (Pol Eko, Poland) equipped with 54 W Master TL5 Ho lamps (Phillips, The Netherlands). Plants were grown under long day conditions: 16 h light and 20°C day/18°C night and 70% humidity.

### Harvesting and treatments with ABA, ET, AUX, KIN, and GA

After 4 weeks of growth, experiments started by harvesting untreated control samples 4 h after the light was turned on. In order to sample the basal first cm of stubble, seedlings were cut at ground level and again 1 cm above ground level, representing mixed sink tissue. Material of 30 seedlings was pooled for each individual biological sample, immediately frozen in liquid nitrogen and stored at −80°C until further use. The remaining plants were either treated with a mock solution consisting of tap water and 0.05% Tween 20 (AppliChem) as a wetting agent to rule out effects caused by the spraying treatment, or a phytohormone solution consisting of tap water, a specific amount of phytohormone and Tween20 as a wetting agent. Five phytohormones were individually applied to seedlings: 10 μM abscisic acid (ABA: Sigma-Aldrich), 10 μM auxin (AUX: 2,4-dichlorophenoxyacetic acid (2,4-D), Sigma-Aldrich), 0.15% ethephon (ET: Sigma-Aldrich), 10 μM gibberellic acid (GA: gibberellic acid potassium salt GA_3_, Sigma-Aldrich), and 10 μM kinetin (KIN: Duchefa, The Netherlands). These phytohormone concentrations were previously found to have an effect on fructan (Morvan et al., 1997; Bravo et al., 1998; Yang et al., 2004), or primary carbohydrate metabolism (Roitsch, 1999; Roitsch and González, 2004; Cardi et al., 2011). Three individual application experiments were carried out per phytohormone. Mock and phytohormone treated samples were harvested 24 and 48 h post treatment, again 4 h after the light was turned on. Prior to extraction and analyses, frozen plant material was pre-homogenized in liquid nitrogen with mortar and pestle, while 0.1% PVPP (Sigma-Aldrich, Munich, Germany) was added to bind phenolic compounds. These mixtures were homogenized to fine powder in a MM400 ball mill (Retsch, GmbH, Germany). Ground plant material was stored at −80°C until further use.

### Extraction and analysis of water soluble carbohydrates

Water-soluble carbohydrates were extracted and analyzed according to Pavis et al. (2001) and Bonfig et al. (2007) using 5 μM mannitol as internal standard. A concentration gradient of 5, 10, 15, and 20 μM for the external standards was used for direct quantification of corresponding carbohydrates: glucose, fructose, sucrose, raffinose (Carl Roth), low DP fructans (1-kestotriose, 6-kestotriose, 6G-kestotriose (kind gifts of Prof. Shiomi), 1,1-kestotetraose, and 1,1,1-kestopentaose (GF5; Wako Chemicals). External standard mixtures containing 5 μM mannitol (for internal standard determination) were purified from charged compounds and particles as described above for carbohydrate extracts from plant material. Glucose, fructose, sucrose, raffinose and the different low DP fructans were separated and quantified via gradient ion chromatography with pulsed amperometric detection on a DIONEX ICS300 HPLC system (Thermo Scientific), equipped with a CarboPac™PA1 column (Thermo Scientific). Peak areas were analyzed using Dionex Chromeleon software (version 6.80, Thermo Scientific). Carbohydrate concentrations were expressed in mg.gFW^−1^. Low DP fructan concentration is the sum of 1-kestotriose, 6-kestotriose, 6G-kestotriose, 1,1-kestotetraose, and 1,1,1-kestopentaose concentrations.

### Extraction of proteins and enzyme assays

For fructan enzyme activities, protein extract was processed as described by Pavis et al. (2001). For carbohydrate enzyme activities, protein extraction was performed according to Jammer et al. (2015). The carbon flux in sink cells regulated by these fructan and carbohydrate enzyme activities is shown in Figure 1. After removal of plant material residues via centrifugation, supernatant was subjected to dialysis according to Bonfig et al. (2007), to remove highly abundant substrates (e.g., sucrose), where needed (Jammer et al., 2015). Protein extracts were snap frozen in liquid nitrogen and stored at −20°C in small aliquots until further use.

Activity of fructan:fructan 1-fructosyltransferase (1-FFT) fructan:fructan 6G-fructosyltransferase (6G-FFT), sucrose:sucrose 1-fructosyltransferase (1-SST), and invertases (cwInv, cytInv, vacInv), was assessed via endpoint assays. Fructan 1-exohydrolase (1-FEH), glucose-6-phosphate dehydrogenase (G6PDH), phosphoglucoseisomerase (PGI), phosphoglucosemutase (PGM), and UDP-glucose dehydrogenase (UGPase) activity was determined in kinetic enzyme assays. Enzymatic activity of carbohydrate enzymes was monitored according to Jammer et al. (2015). Fructan enzyme activities were monitored via product abundance (1-kestotriose, 1,1-kestotetraose, 6G-kestotriose) upon incubation (1-SST, 1-FFT, 6G-FFT assays, respectively). Activity of invertases and all enzymes measured via kinetic assays was determined as described by Jammer et al. (2015). Products of endpoint activity assays for fructan enzymes were detected and quantified as described above for WSCs. Samples were run in triplicate with blanks, omitting substrate and controls, omitting enzyme extract. Specific enzyme activity was calculated within the linear range of substrate conversion, corrected for background from blanks or controls and expressed in nkat.gFW^−1^.

For combined 1-FFT and 6G-FFT activity assay, 25 μL of dialyzed enzyme extract was incubated with 50 mM sucrose and 50 mM 1-kestotriose in 80 mM potassium phosphate buffer pH 5.5, at 30°C for 120 min For 1-SST activity 25 μL dialyzed extract were incubated with 50 mM sucrose in 50 mM potassium phosphate buffer pH 5.7, at 30°C for 60 min enzymatic activity was stopped by heating the mixtures to 99°C for 3 min After centrifugation for 10 min at 4°C, samples were transferred into fresh tubes and diluted 10-fold including mannitol as an internal standard at a final concentration of 5 μM (modified from Pavis et al., 2001) and purified from charged compounds and particles as described by Bonfig et al. (2007). 1-FFT activity was calculated from the amount of 1-nystose generated upon incubation with sucrose and 1-kestose. 6G-FFT activity was calculated from the amount of 6G-kestotriose generated upon incubation with sucrose and 1-kestotriose. 1-SST activity was calculated from the amount of 1-kestotriose generated upon incubation with sucrose.

For 1-FEH activity assay, 10 μL of dialyzed enzyme extract were incubated with 1 mM EDTA set to pH 8 with 10 M NaOH, 2 mM MgCl_2_(Carl Roth, Germany), 50 mM 1-kestotriose (1-K; Wako Chemicals GmbH, Germany), 1.3 mM ATP (AppliChem, Germany), 0.5 mM NAD, 5 mM DTT (Carl Roth), 0.4 U glucose-6-phosphate dehydrogenase from *Leuconostoc mesenteroides* (G6PDH EC 1.1.1.49, Oriental Yeast, Japan), 0.672 U hexokinase from yeast (HK 2.7.1.1, Roche, Germany) and 0.56 U PGI from yeast (Roche), added up to a total assay volume of 160 μL with 80 mM citrate phosphate buffer, pH 5.5 as described in Gasperl et al. (2015: accompanying paper submitted to FiPS). Invertases, G6PDH, PGI, PGM, and UGPase activities were assayed as specified for perennial ryegrass samples by Jammer et al. (2015), using a 2.5 μL aliquot for cwInv and vacInv activity, a 5 μL aliquot for cytInv activity and a 10 μL aliquot for G6PDH, PGI, PGM and UGPase activities, respectively.

### RNA extraction and cDNA synthesis

Total RNA was extracted from 100 mg frozen, ground stubble, using the RNeasy plant mini kit from Qiagen (Hilden, Germany), according to the manufacturer's instructions. The mRNA concentration was determined with a NanoDrop UV/Vis spectrophotometer (Thermo Scientific), measuring absorbance at 260 nm in 1 μL of RNA extract. cDNA was synthesized from 1 μg mRNA per 20 μL reaction via RT-PCR with RevertAid Reverse Transcriptase from *E. coli* and Oligo(dT)18 primers (Thermo Scientific / Fermentas, Waltham, MA, USA), according to the manufacturer's instructions.

### Quantitative RT-PCR analysis

Expression levels of target and housekeeping genes were determined in 96-well format microplates on a Chromo4™ Four-Color Real-Time PCR system (Bio-Rad, Marne-la-Coquette, France), using the 2x iQ SybrGreen Supermix (Bio-Rad). Each reaction consisted of 7.5 μL 2x SybrGreen Supermix, 0.75 μL 10 μM sense primer (see specific primers listed below), 0.75 μL 10 μM antisense primer, 2 μL sterile double distilled water (Milli-Q®, Merck Millipore, Merck, Darmstadt, Germany) and 4 μL of 100-fold (or 10-fold) diluted cDNA sample as a template. Three biological samples of control or treated plants, respectively, were run in duplicate. Sterile double distilled water was used as a blank to determine background e.g., from primer dimerization. Since DNAse digestion was not applied during the RNA extraction, the quantitative RT-PCR was also run for each sample prior to cDNA synthesis (negative controls) to take possible genomic DNA contamination into account. The quantitative RT-PCR program included the following steps: 3 min at 95°C, 40 cycles of: 10 s at 95°C, and 40 s at 60°C. The melting curve went from 55 to 95°C in 1°C steps per second. Most stable housekeeping genes for stubble were tested from a set of perennial ryegrass genes recommended by Martin et al. (2008). After pre-analysis of most stable housekeeping genes in ryegrass seedling stubble, *eEF-1*α and *GAPDH* were chosen as housekeeping genes for all expression level analyses. Chromo4 software was used to calculate C(t) levels. For calculation of fold changes of RNA transcripts, the comparative threshold cycle method was used according to Livak and Schmittgen (2001). Values were rescaled for housekeeping gene abundance and normalized to levels of untreated control plants. Primers for target genes (*Lp1-SST, Lp6-SFT, Lp6G-FFT, Lp1-FEH, Lp6-FEH, LpSUT1*) used for qPCR analyses are listed in Table 1.

**Table 1:** **Primers used for quantitative RT-PCR analysis of perennial ryegrass cDNA**.

**Accession number**	**Gene**	**Primer**	**Primer sequence**
EU168438.1	*eEF-1α*	Forward	5′-ggctgattgtgctgtgctta-3′
	*eEF-1α*	Reverse	5′-ctcactccaagggtgaaagc-3′
EF463063.1	*GAPDH*	Forward	5′-catcaccattgtccaacg-3′
	*GAPDH*	Reverse	5′-aaccttcaacgatgccaaac-3′
DQ016297	*Lp1-FEH*	Forward	5′-cacatcaaagctacatggtt-3′
	*Lp1-FEH*	Reverse	5′-ccactggataaactctggac-3′
EU219846	*Lp6-FEH*	Forward	5′-tgcctaccactcccagtct-3′
	*Lp6-FEH*	Reverse	5′-atgacaggctgatcaccagg-3′
AY245431	*Lp1-SST*	Forward	5′-gccaggtcatcctgctctac-3′
	*Lp1-SST*	Reverse	3′-ccggcatgagctcgtagtt-3′
AB125218	*Lp6G-FFT*	Forward	5′-tctcaactcttcggacatcga-3′
	*Lp6G-FFT*	Reverse	5′-tacatgtcgtcagccaagaag-3′
AF494041.1	*Lp6-SFT*	Forward	5′-cagcttctgcaacgacga-3′
	*Lp6-SFT*	Reverse	5′ccttaaccatgacggtctcg-3′

### Statistical analysis

The mean and standard error (SE) was calculated from two or three biological samples for each of the three independent application experiments. Statistical analyses of data were conducted using R version 3.0.1 (R Core Team, 2013). Data normality and variance homo-geneity were verified using the Shapiro-Wilk's test (99%) and Bartlett's test (99%), respectively. Raw data were transformed (log, square, square root or inverse transformations) when needed. Data were subjected to analysis of variance (One-way ANOVA) between the five treatment modalities (time 0; time 24 h mock treated plants; time 24 h phytohormone treated plants; time 48 h mock treated plants; time 48 h phytohormone treated plants). When there was a significant (*P* ≤ 0.05) effect of treatment, a Tukey's test was used for comparison between the five conditions and differences between means (*P* ≤ 0.05) were indicated by different letters above each bar.

## Results

**Figure 2:**
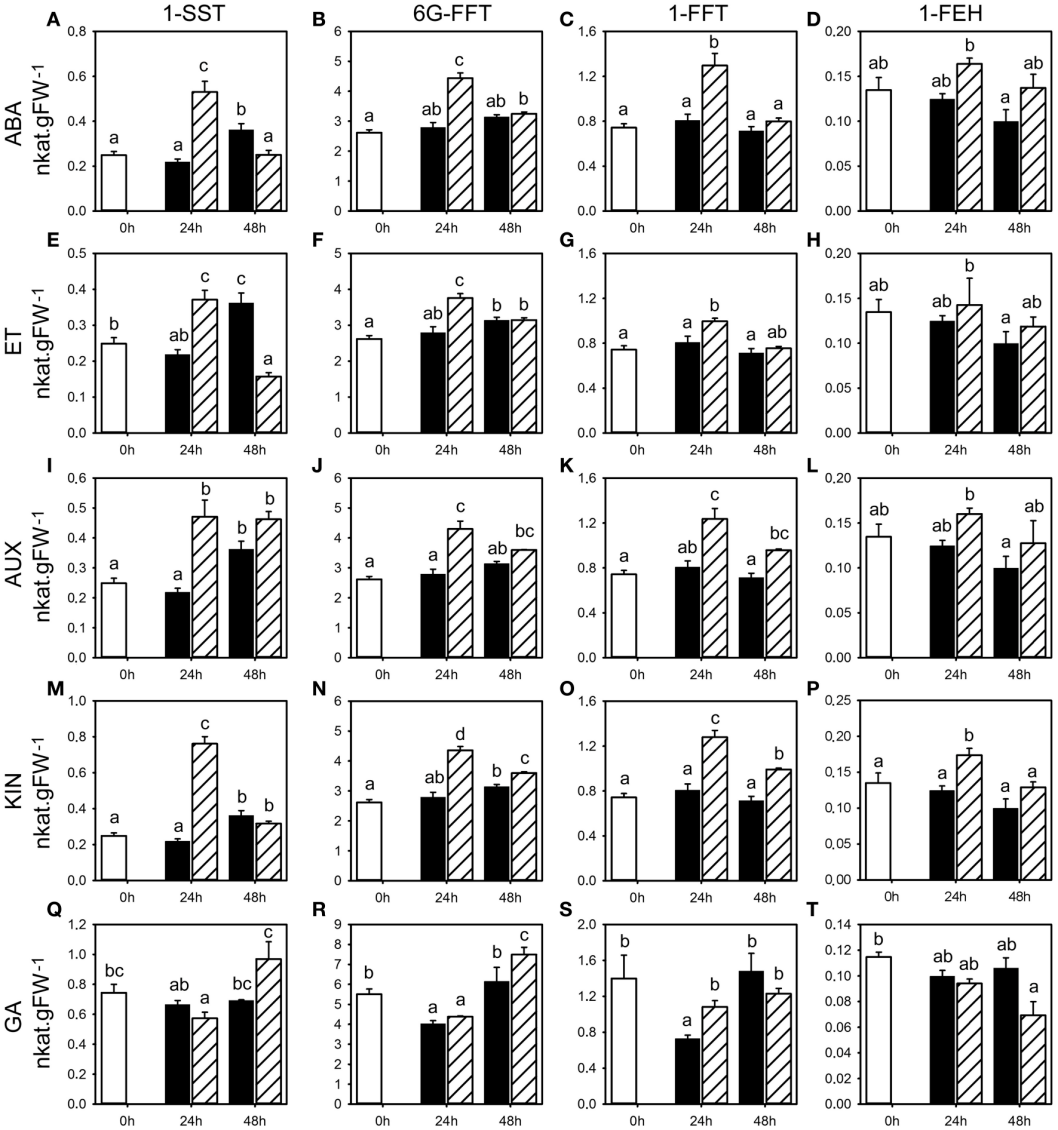
**Activities for enzymes involved in fructan metabolism following phytohormone treatments**. Enzyme activities of 1-SST, 6G-FFT, 1-FFT, and 1-FEH (nkat.gFW^−1^) in the first basal cm of stubble of 4-week old perennial ryegrass seedlings 24 or 48 h post ABA **(A–D)**, ET **(E–H)**, AUX **(I–L)**, KIN **(M–P)**, or GA **(Q–T)** treatment, compared to untreated controls (0 h). Open bars, untreated controls; closed bars, mock treatment; dashed bars, phytohormone treatment. Values represent the mean of three biological replicates ± SE. Different letters indicate statistical significance at *p* < 0.05. Representative examples are shown from one of the three experiments.

**Figure 3:**
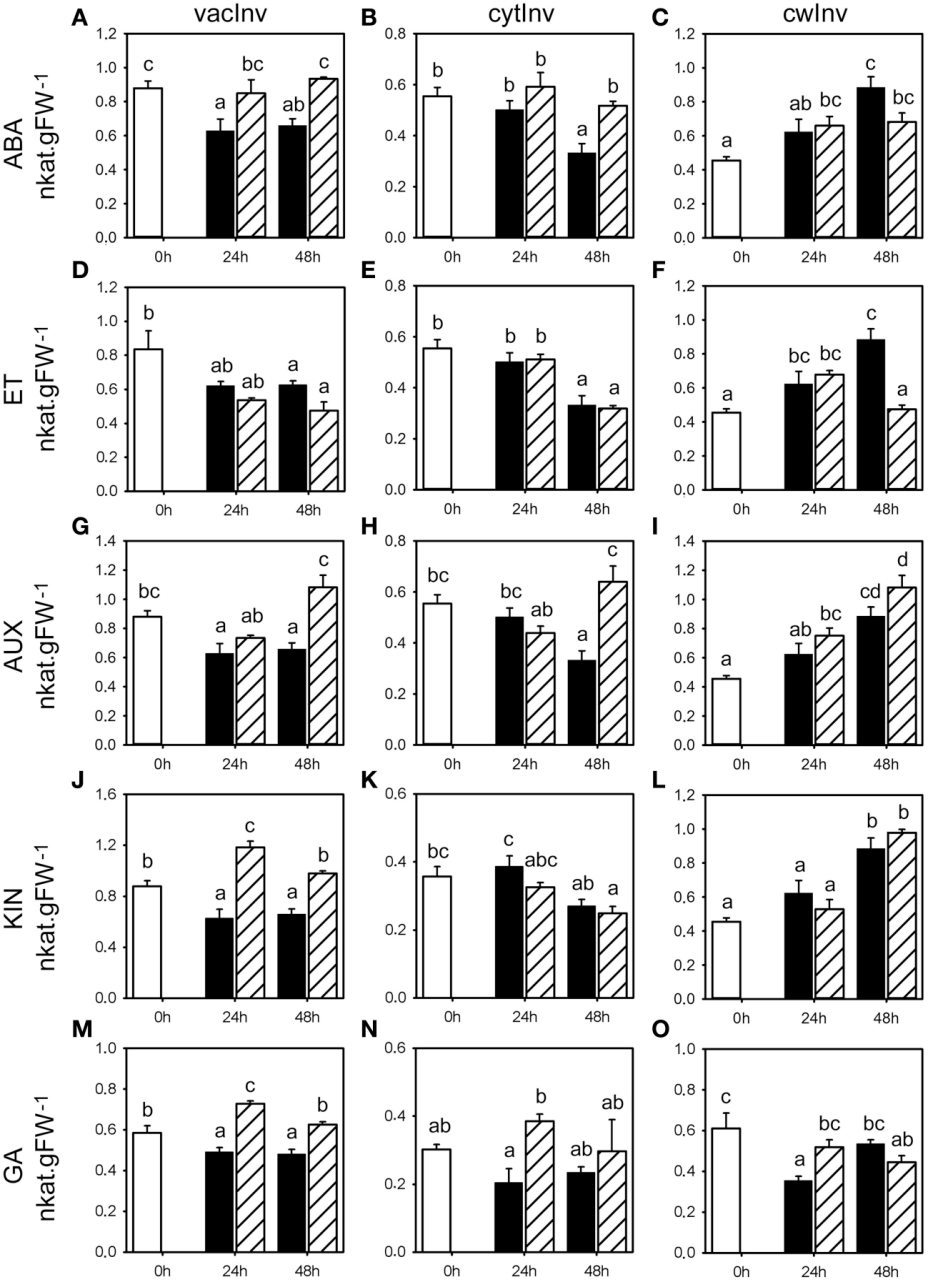
**Invertase enzyme activities following phytohormone treatments**. Enzyme activities of vacInv, cytInv, and cwInv (nkat.gFW^−1^) in the first basal cm of stubble of 4-week old perennial ryegrass seedlings 24 or 48 h post ABA **(A–C)**, ET **(D–F)**, AUX **(G–I)**, KIN **(J–L)**, or GA **(M–O)** treatment, compared to untreated controls (0 h). Open bars, untreated controls; closed bars, mock treatment; dashed bars, phytohormone treatment. Values represent the mean of three biological replicates ± SE. Different letters indicate statistical significance at *p* < 0.05. Representative examples are shown from one of the three experiments.

**Figure 4:**
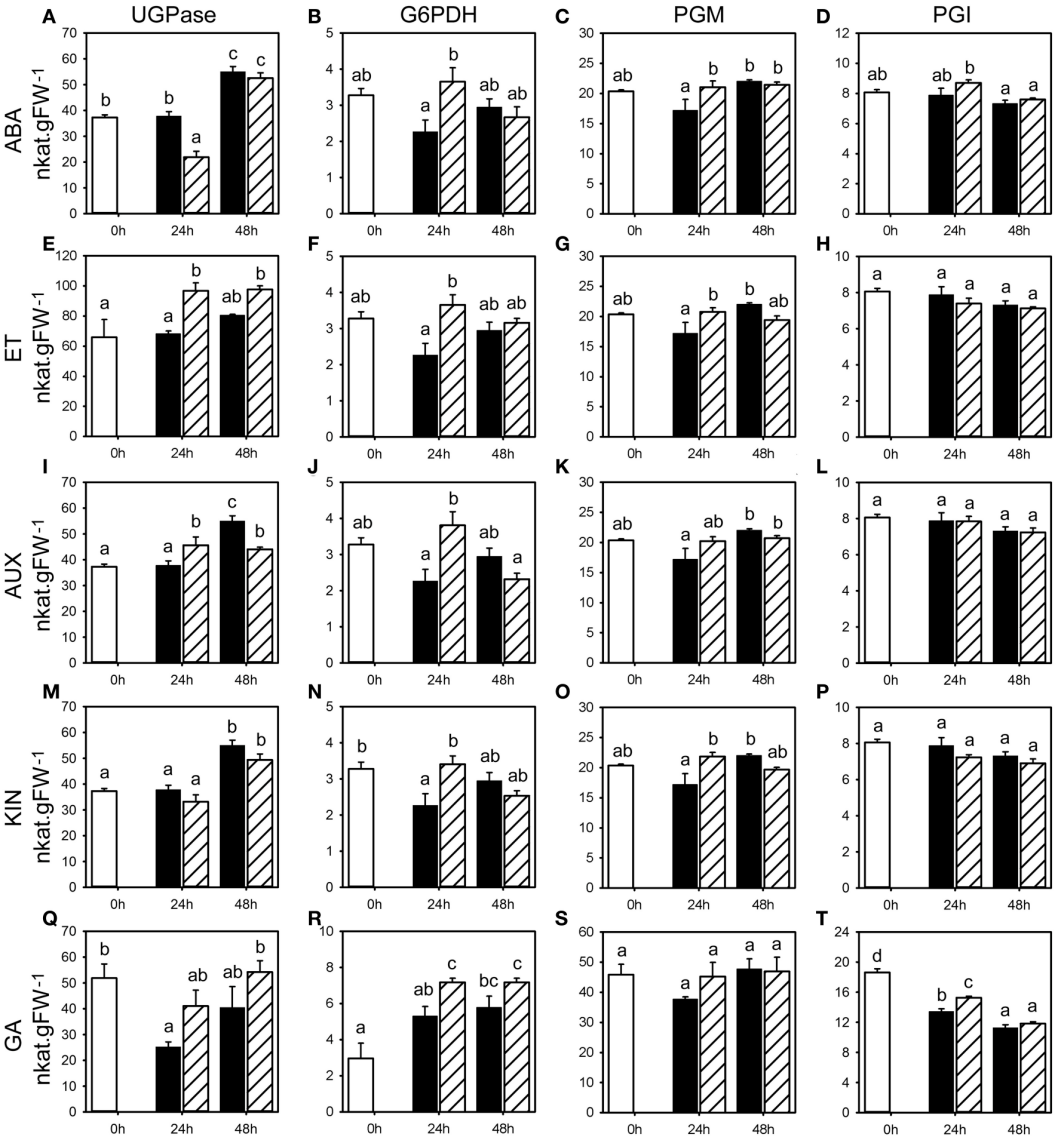
**Activities for enzymes involved in primary carbohydrate metabolism following phytohormone treatments**. Enzyme activities of UGPase, G6PDH, PGM, and PGI (nkat.gFW^−1^) in the first basal cm of stubble of 4-week old perennial ryegrass seedlings 24 or 48 h post ABA **(A–D)**, ET **(E–H)**, AUX **(I–L)**, KIN **(M–P)**, or GA **(Q–T)** treatment, compared to untreated controls (0 h). Open bars, untreated controls; closed bars, mock treatment; dashed bars, phytohormone treatment. Values represent the mean of three biological replicates ± SE. Different letters indicate statistical significance at *p* < 0.05. Representative examples are shown from one of the three experiments.

**Figure 5:**
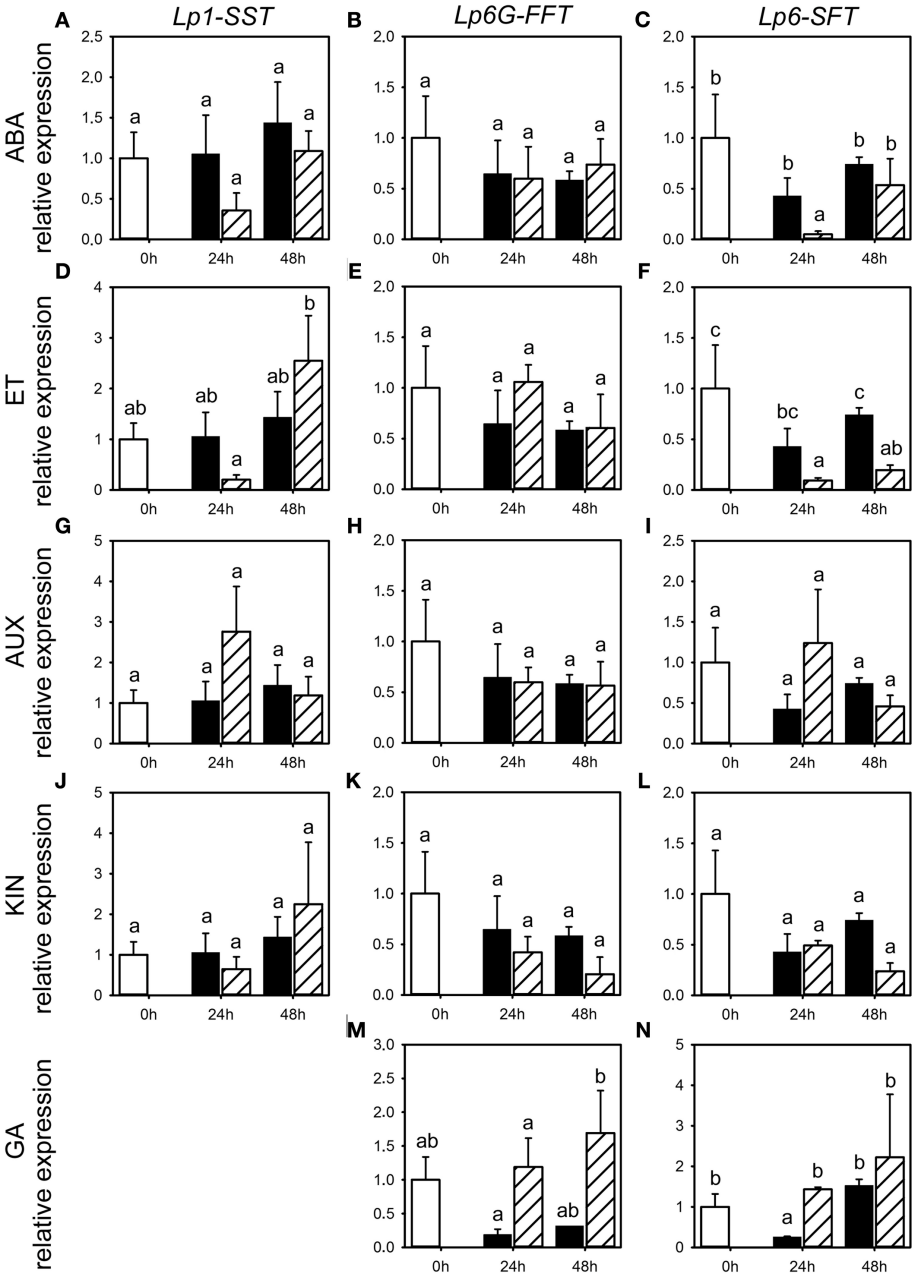
**Expression of *FTs* following phytohormone treatments**. Quantitative RT-PCR analysis of *Lp1-SST, Lp6G-FFT*, and *Lp6-SFT* expression in stubble of 4-week old perennial ryegrass seedlings 24 or 48 h post ABA **(A–C)**, ET **(D–F)**, AUX **(G–I)**, KIN **(J–L)**, or GA **(M,N)** treatment. Open bars, untreated controls; closed bars, mock treatment; dashed bars, phytohormone treatment. Transcript levels were normalized to untreated controls (expression at 0 h). Values represent the mean of three biological replicates ± SE. Different letters indicate statistical significance at *p* < 0.05.

**Figure 6:**
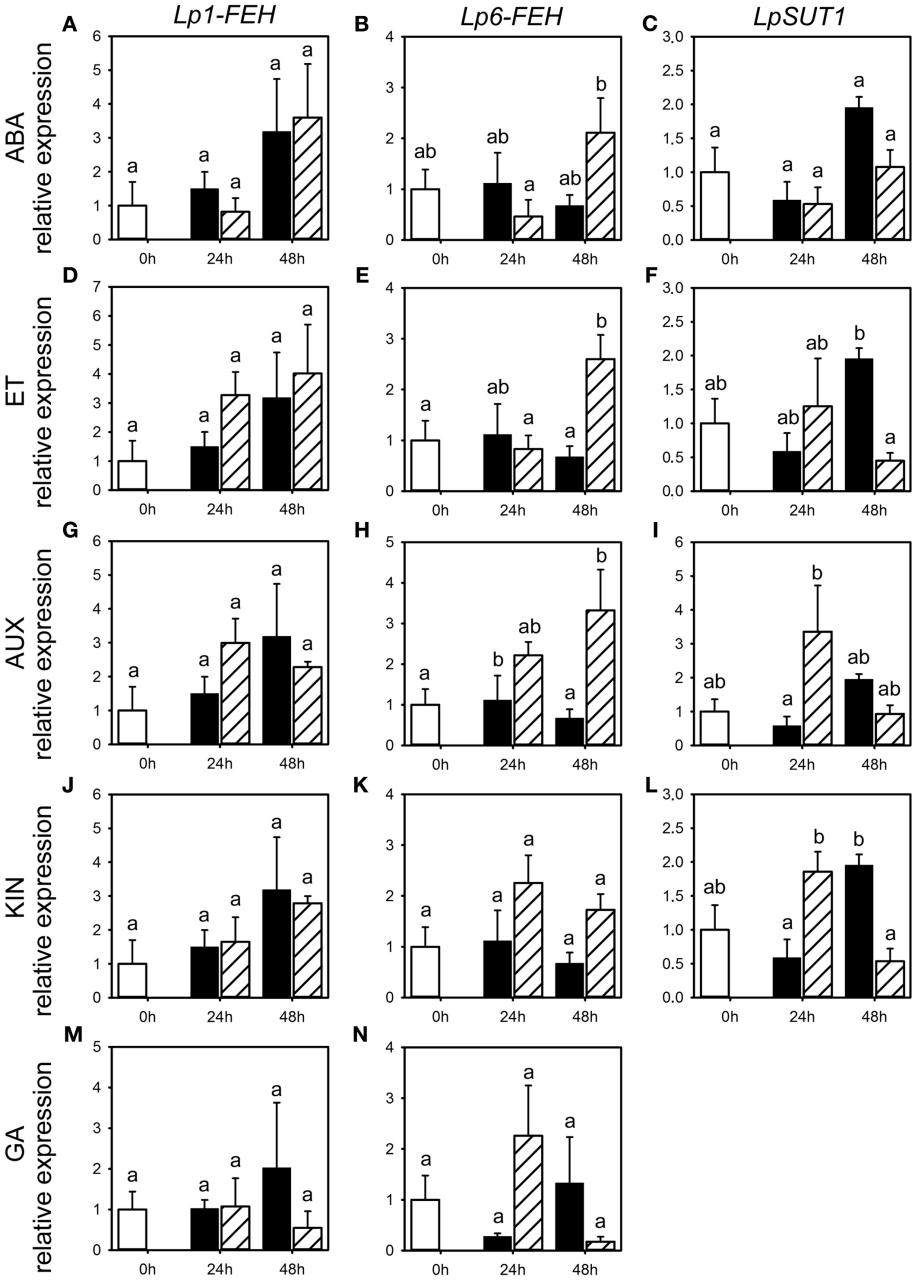
**Expression of *FEHs* and the *SUT1* sucrose transporter following phytohormone treatments**. Quantitative RT-PCR analysis of *Lp1-FEH, Lp6-FEH*, and *LpSUT1* expression in stubble of 4-week old perennial ryegrass seedlings 24 or 48 h post ABA **(A–C)**, ET **(D–F)**, AUX **(G–I)**, KIN **(J–L)**, or GA **(M,N)** treatment. Open bars, untreated controls; closed bars, mock treatment; dashed bars, phytohormone treatment. Transcript levels were normalized to untreated controls (expression at 0 h). Values represent the mean of three biological replicates ± SE. Different letters indicate statistical significance at *p* < 0.05.

**Figure 7:**
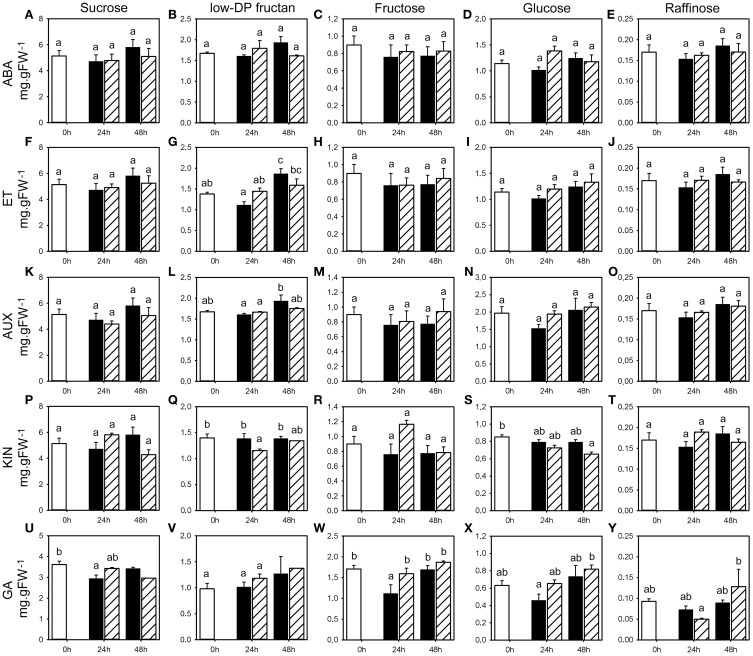
**WSC concentrations following phytohormone treatments**. Concentrations of sucrose, low-DP fructans fructose, glucose, and raffinose (mg.gFW^−1^) in the first basal cm of stubble of 4-week old perennial ryegrass seedlings 24 or 48 h post ABA **(A–E)**, ET **(F–J)**, AUX **(K–O)**, KIN **(P–T)**, or GA **(U–Y)** treatment, compared to untreated controls (0 h). Open bars, untreated controls; closed bars, mock treatment; dashed bars, phytohormone treatment. Values represent the mean of three biological replicates ± SE (except **V** 48 h GA: one sample). Different letters indicate statistical significance at *p* < 0.05. Representative examples are shown from one of the three experiments.

We studied the effect of the classical five classes of phytohormones on fructan metabolism by determining the according enzyme activities and gene expression levels, and WSC levels, using seedlings grown on vermiculite in growth chambers to mimic as close as possible natural conditions. Phytohormones were applied by single pulse treatments using spray application to minimize pleiotropic effects related to long exposure of non-physiological concentrations, in three independent experiments for each phytohormone treatment. Since these spray applications by itself can have an effect on fructan metabolism by eliciting stress responses, the effect of phytohormones 24 and 48 h following application was compared to the corresponding mock treatments. Gene expression, fructan, and primary carbohydrate enzyme activities and WSC levels were followed in the basal first cm of the stubble shown to contain the highest fructan levels. Representative examples that reflect the overall change for the respective parameters in all three experiments are shown for the activities of enzymes involved in fructan (Figure 2) and primary carbohydrate metabolism (Figures 3, 4), and WSC levels (**Figure 7**). Most representative examples were shown from the third experiment. Because of the weak changes in fructan enzyme activities and WSC levels, the gene expression (Figures 5, 6) was only assessed for the third experiment.

### Effects of phytohormone treatments on fructan enzyme activities

The analysis of fructan enzyme activities showed that ABA and ET treatment generally caused a transient increase in the activity of fructan biosynthesis enzymes (1-SST, 6G-FFT, 1-FFT) compared to the untreated controls and mock treatments (Figure 2, Supplemental Tables 1, 2). A significant change in activity was shown for 1-SST (Figure 2A, in two experiments with up to 140% increase) and 1-FFT (Figure 2C, one experiment with 61% increase) by ABA, and 1-SST (Figure 2E, two experiments with up to 70% increase), 6G-FFT (Figure 2F, two experiments) and 1-FFT (Figure 2G, one experiment) by ET. At 48 h post treatment, mock treated plants showed a positive trend in 1-SST activity, suggesting a stress response elicited by the spraying treatment, whereas in ABA and ET treated plants, 1-SST activity was below (ET) or remained at the level of control plants (ABA). Consequently, ABA and ET treatment led to a clear decrease in 1-SST activity compared to mock treatment 48 h post treatment, with significant change in two experiments (Figures 2A,E, Supplemental Tables 1, 2). ABA and ET treatment had no major impact on the degrading 1-FEH activity at 24 and 48 h (Figures 2D,H, Supplemental Tables 1, 2).

AUX and KIN treatment also resulted in generally increased activities for all fructan biosynthesis enzymes (1-SST, 6G-FFT, 1-FFT) compared to untreated controls and mock treatments (Figure 2, Supplemental Tables 3, 4). The observed increases in activities were most pronounced and thus transiently increased at 24 h compared to 48 h for all three enzymes in response to both AUX and KIN in two out of the three experiments. Within the various data, the most significant changes were observed for 1-SST (Figures 2I,M) in two experiments (AUX 24 h and KIN 24 h, with up to 116 and 248% increase, respectively), 6G-FFT (Figures 2J,N) in two experiments (AUX 24 h and KIN 24 h with up to 100% increase), and 1-FFT (Figures 2K,O) in two experiments (AUX 24 h and KIN 24 h with up to 230% increase). No general regulatory pattern was evident for 1-FEH with the exception of KIN treatment, that caused a significant increase in 1-FEH activity at 24 h in two experiments (Figures 2L,P).

The effect of GA treatment was less pronounced although generally higher activities were again observed at 24 h. Mock treatment led to a transient negative trend in 6G-FFT and 1-FFT enzyme activities compared to untreated controls, while GA treatment resulted in a late increase of activities (Figures 2R,S), with significant change in two experiments (Supplemental Table 5) at 24 h for 1-SST (up to 74% higher) and also 1-FFT, whereas the increase was significant for 6G-FFT in only one experiment (Figure 2R). GA treatment caused lower levels in 1-FEH activity compared to untreated controls at 48 h post treatment (Figure 2T), with significant change in two experiments.

In summary, pulse treatments by five phytohormones had generally only minor effects on activities for the enzymes involved in fructan metabolism that were at best significant in two out of three experiments (Supplemental Figure 1, Supplemental Tables 1–5). The most pronounced effects were a transient increase of the three biosynthetic activities 1-SST, 1-FFT, and 6G-FFT by the phytohormone treatments, most pronounced for AUX and KIN. Generally minor effects were evident on the catabolic 1-FEH activity with an observed increase of the activity in response to KIN at 24 h and a decrease by GA at 48 h.

### Effects of phytohormone treatments on enzyme activities that regulate the sucrose pool

To address the relation between fructan and primary carbohydrate metabolism, we evaluated how the activity of key enzymes involved in carbohydrate metabolism that are involved in the availability of sucrose was affected by the various phytohormone treatments. Since fructan synthesis requires sucrose and sucrose levels influence the activity of FTs and FEHs, we studied seven key enzymes involved in primary carbohydrate metabolism that affect the sucrose pool (cytInv, cwInv, vacInv, G6PDH, PGI, PGM, and UGPase). The activities of vacInv, that competes with the fructan biosynthetic enzymes for the substrate sucrose, has been compared with the activities for the extracellular, cell wall bound cwInv and cytoplasmic cytInv. In addition, the activities of PGI and PGM (glycolysis), G6PDH (oxidative pentosphosphatecycle) and UGPase (resynthesis of sucrose) were determined. A novel experimental platform for the measurement of all these activities from a single extract in miniaturized assays in microtiter plates has been used (Jammer et al., 2015).

ABA treatment caused an increase in vacInv and cytInv activities, because the corresponding mock treatments led to a negative trend compared to the untreated controls, with significant change in vacInv activity (Figure 3A) in two experiments at 24 and 48 h, and cytInv activity (Figure 3B) at 48 h in two experiments (Supplemental Table 1). The effect on cwInv (Figure 3C) was inconclusive between the three experiments. No distinct effects of ABA could be observed on the activities of UGPase, G6PDH, PGM, and PGI (Figures 4A–D). In contrast to the effect on fructan enzyme activities, the effect of ET on the invertase activities differed from ABA treatment and resulted in inconsistent effects on vacInv, cytInv, and cwInv (Figures 3D–F, Supplemental Table 2). PGM and PGI activities were not affected (Figures 4G,H). ET resulted in elevated activity levels of UGPase (Figure 4E, with significant increase in two experiments at 24 and 48 h) and G6PDH (Figure 4F), only at 24 h in two experiments).

AUX had only minor effects on the measured enzyme activities (Figure 3; Supplemental Table 3). The most pronounced effects were on cytInv and UGPase activities. cytInv activity was elevated at 48 h, with a significant increase in two experiments by maximal 92% and UGPase activity was significantly elevated in two experiments at 24 h by at least 30% (Figures 3H, 4I). The most pronounced effect of KIN was on the activities on two invertase isoenzymes. cwInv was elevated significantly at 48 h in two experiments by up to 85% (Figure 3L) and vacInv was elevated significantly at 24 h in one experiment by 74% and at 48 h in two experiments by at least 30% (Figure 3J). In contrast, KIN had only a minor effect on the activities of all the other measured enzymes (Figures 3, 4, Supplemental Table 4).

GA treatment primarily resulted in an increase in activities at 24 h with a significant effect in two experiments on the activities of vacInv (at least 30%), cytInv (up to 89%), UGPase (at least 30%), and G6PDH (at least 30%) (Figures 3M,N, 4Q,R). The only significant effect at 48 h in two experiments was on vacInv. The effect on cwInv was inconclusive between the three experiments (Supplemental Table 5) although an increase by 209% was observed in one experiment at 48 h (Supplemental Table 5).

In summary, the analysis of the seven enzymes that are involved in primary carbohydrate metabolism showed that predominantly vacInv, cytInv, cwInv, UGPase, and G6PDH activities were affected at the later time point with significant regulation in two experiments by the phytohormone treatments (Supplemental Figure 1, Supplemental Tables 1–5). In contrast, the phytohormone treatments caused small changes (<30%) in PGM activities, while PGI activity was virtually not affected at all.

### Effects of phytohormone treatments on gene expression of fructan enzymes and sucrose-1-transporter

Because of the minor and occasionally inconsistent effect of the phytohormone treatments on the activity of enzymes involved in fructan metabolism, we assessed the effect of phytohormones on transcript levels for FTs (*Lp1-SST, Lp6-SFT* and *Lp6G-FFT*) and fructan degrading enzymes (*Lp1-FEH* and *Lp6-FEH*) by qRT-PCR for the third experiment only (Figures 5, 6). Compared to the corresponding mock treatments, *Lp6-SFT* expression level was significantly decreased 24 h following ABA treatment (Figure 5C) and *Lp1-SST* expression level tended to decrease (Figure 5A). In contrast, *Lp1-FEH* level (Figure 6A) was increased 48 h following ABA treatment, but also by the corresponding mock treatment. In addition, *Lp6-FEH* level (Figure 6B) was also increased 48 h following ABA treatment, but only significant when compared to the expression 24 h following ABA treatment. The *Lp1-SST* level (Figure 5D) was transiently decreased 24 h following ET treatment. *Lp6-SFT* expression (Figure 5F) was significantly reduced 24 and 48 h following ET treatment. *LpFEH* expression levels (Figures 6D,E) were increased 48 h following ET treatment, but this increase was significant only for *Lp6-FEH* (Figure 6E).

AUX treatment led to a transient, but not significant, increase in *Lp1-SST* and *Lp6-SFT* expression (Figures 5G,I) 24 h following treatment, compared to the corresponding mock controls, while *Lp6G-FFT* expression was not affected (Figure 5H). *Lp6-FEH* expression (Figure 6H) was increased 24 and 48 h (significant) following AUX treatment compared to the corresponding controls. *Lp6-SFT* and *Lp6G-FFT* expression generally decreased following KIN treatment (Figures 5K,L), but not significantly. Similar to AUX treatment, *Lp6-FEH* expression was increased, but not significantly, 24 and 48 h following KIN treatment compared to the corresponding controls (Figure 6K).

GA treatment increased *Lp6G-FFT* levels 48 h following treatment, compared to the corresponding mock control (Figure 5M), while *Lp1-FEH* expression tended to be reduced compared to the corresponding mock controls 48 h following GA treatment (Figure 6M).

In general, *LpSUT1* level was transiently increased 24 h following application of ET, AUX or KIN (Figures 6F,I,L) with significant modification compared to mock for AUX and KIN. In contrast, *LpSUT1* level was lower 48 h following application of ABA, ET, AUX, or KIN (Figures 6C,F,I,L) compared to mock treatment, with significant modifications for ET and KIN, but was not different from the untreated controls.

In summary, the phytohormone treatments resulted in a fast but weak effect on the transcriptional regulation, similar to the predominantly minor changes observed for activities of enzymes involved in fructan metabolism (Figure 2).

### Effects of phytohormone treatments on carbohydrate content

Generally, weak changes, in a number of cases significant, were detected for the expression and activities of the enzymes involved in fructan metabolism, as well as key enzymes involved in primary carbohydrate metabolism. The analyses of transcript levels and enzyme activities were complemented by the analysis of the impact of the treatment by the five phytohormones on WSC content. None of the treatments resulted in obvious or significant changes in WSC content during the first 48 h following phytohormone treatments (Figure 7, Supplemental Tables 6–10), except for lowDP fructan. Low DP fructan concentration tended to increase at 24 h post ABA, ET and AUX treatment compared to untreated control and mock treatment (Figures 7B,G,L) with significant changes in only one experiment at 24 h (ABA and ET), but decreased following KIN (Figure 7Q) with significant change only in one experiment at 24 h. At 48 h post ABA, ET, AUX, and KIN treatment, low DP fructan concentration remained at the same level when compared to untreated control and tended to be lower than in mock treated plants. Sucrose, hexose, and raffinose levels (Figure 7) were only slightly affected by phytohormone treatments with minor trends shared between the three experiments.

## Discussion

To assess the possible short term hormonal regulation of fructan metabolism we have carried out an extensive analysis of the impact of all five classical phytohormones ABA, ET, AUX, KIN, and GA on the activities of enzymes involved in the biosynthesis (1-SST, 1-FFT, 6G-FFT) and degradation (1-FEH), as well as the corresponding transcript and metabolite levels 24 and 48 h after a single pulse treatment. These data were complemented by testing also the impact of the phytohormone treatments on the activities of seven key enzymes of primary carbohydrate metabolism. Our data provide a valuable expansion of the knowledge on the influence of phytohormones on the regulation of fructan metabolism in perennial ryegrass, since only the effect of GA has been studied before *in planta* in this plant species (Morvan et al., 1997), but this was done simultaneously with uniconazole, an inhibitor of plant growth regulator synthesis. Previous studies investigating hormonal effects on fructan metabolism in various plant species showed strong effects on fructan accumulation and corresponding enzyme activities and/or gene expression (Bravo et al., 1998; Kerepesi et al., 2004; Yang et al., 2004; Ruuska et al., 2008; Suárez-González et al., 2014). However, these studies often used tissue culture approaches and/or liquid medium, with long exposure and continuous treatment of phytohormones, resulting in experimental conditions that were far from the natural growth situation. Based on the very limited current knowledge on hormonal regulation of fructan metabolism in higher plants, specifically in perennial ryegrass, the primary goal of the present study was to obtain a very first and comparative insight into the impact of the treatment by all five classical phytohormones on fructan metabolism. Within the ambition of a very broad and comprehensive approach, determining different complementary parameters within the same experimental system, we had to choose single concentrations of the five different phytohormones applied to limit the number of experimental variables.

Based on the general observation that the effect of exogenous phytohormones depends to a large extent on the concentration applied and within the constraint and experimental limitation of testing only a single phytohormone concentration for each of the different phytohormones compared, the concentrations were carefully selected based on literature data. The chosen concentration of ABA, KIN, and GA3, 10 μM each, were previously shown to regulate fructan active enzymes (Lalk and Dörffling, 1985; Bravo et al., 1998; Yang et al., 2004; Ruuska et al., 2008; Suárez-González et al., 2014), GA3 even in ryegrass (Morvan et al., 1997). Exogenous applications of these three phytohormones and also of ET and the auxin IAA in the applied concentration were shown to be biological active also in other grasses (Yin and Quinn, 1992; Brock et al., 1994; Goupil et al., 1998; O'Mahony and Oliver, 1999; Zhang and Schmidt, 2000; Bais et al., 2001; Xu and Huang, 2009; Yang et al., 2012). However, also in other grasses only a very limited number of studies has been published on exogenous phytohormone application. The chosen concentrations were also typically and successfully used in numerous studies on exogenous applications of phytohormones in a variety of other plant species. We have specifically aimed to address the influence of phytohormones on the regulation of fructan metabolism using experimental conditions that are closer to the natural growth situation, using seedlings grown in a solid substrate in growth chambers and single pulse phytohormone treatments, rather than continuous high, non-physiological phytohormone levels. Although we detected much weaker effects of exogenous phytohormone treatment on fructan metabolism compared to earlier studies, relatively fast effects within 2 days after the pulse treatment on the activities of enzymes involved in fructan and primary carbohydrate metabolism, and expression of the corresponding *FT* and *FEH* genes as well as the *LpSUT1* gene encoding a sucrose transporter could be demonstrated. In general, the changes in the activities of the tested enzymes were observed with variability of the observed effects in independent experiments (Supplemental Figure 1, Supplemental Tables 1–10). This may be due to the single treatment of seedlings that was employed to avoid non-physiological effects.

The most pronounced effects were an early increase of all the fructan synthesis enzymes 1-SST, 1-FFT, 6G-FFT by KIN and AUX, with a significant elevation of the activities for all three activities in two experiments by up to 143% by AUX and by up to 248% by KIN, followed by lower though elevated activity levels at 48 h. Likewise, but less consistent within the three tested enzymes, also ABA, ET, and GA resulted in elevated activity levels of the biosynthetic enzymes at the early time point, with a most pronounced effect on 1-SST and 6G-FFT activities (ABA, ET), and 1-SST and 1-FFT (GA), respectively. In contrast, the various phytohormones had only a minor effect on the activity of the fructan degrading enzyme 1-FEH with a moderate decrease by GA by at least 30% at 48 h as the strongest effect. There was no apparent co-regulation of the activity for any of the seven tested enzymes of primary carbohydrate metabolism, whereas the effects on the enzyme activities were also generally small but the most pronounced effect typically apparent only at the later time point of 48 h. Consistent with the only minor effect on the fructan metabolism enzyme activities no major and significant effects of the pulse treatment by the five phytohormones were observed for the WSC content. Also in other studies a very weak correlation between enzyme activities and metabolite levels has been observed. In a complex network analysis of enzyme activities and metabolite levels and their relationship to biomass with a large panel of *Arabidopsis* accessions (Sulpice et al., 2010) only very few correlations between enzyme activities and metabolites were observed. Within a matrix containing 62 metabolites and 37 enzymes, allowing 2294 pair-pair correlations, there was only one significant correlation when the traits are compared on a protein basis.

The pulse treatment by the five phytohormones had fast, but only small effects, on the transcript levels encoding the biosynthetic genes *Lp1-SST, Lp6-SFT, Lp6G-FFT*, the two degrading enzymes *Lp1-FEH* and *Lp6-FEH* and the sucrose transporter *LpSUT1*. Thus, there is no correlation to the measured corresponding enzyme activities. This is in agreement with the general observation that transcripts and protein abundance often poorly correlate (Stitt and Gibon, 2014) and that the correlations are even worse if it comes to enzyme activities. Since the determination of the abundance of transcripts is widely used as proxy to assess the function of enzymes involved in metabolic processes, including for fructan metabolism (Abeynayake et al., 2015), the observed discrepancy demonstrates the importance of the determination of enzyme activities to obtain insight into the physiological relevance. Between the transcript level and enzyme activities various levels of posttranscriptional and posttranslational regulation are operational and notably affected also by various external factors and internal regulatory mechanisms. Thus, the steady state levels of enzyme activities are more relevant to the physiological traits and ultimately the plant phenotype.

The phytohormone treatments appeared to have no major short term effect on WSC levels including sucrose, the fructan precursor, in perennial ryegrass. Bravo et al. (1998) reported a significant increase in soluble sugars in barley leaves after 4 days of cold acclimation or 5 days of ABA treatment, and Hisano et al. (2008) similarly found changes after 4 days of cold treatment. This suggests that although the phytohormones were exogenously applied, a 2 days period is too short for the detected enzyme activity changes to take effect for significant changes in WSC levels. We successfully employed similar pulse-spray applications for phytohormone treatments of tomato fruits (Albacete et al., 2014) and induction of gene expression (Hyun et al., 2014). Therefore, the absence of strong and significant short effects of the phytohormone treatments on the analyzed enzyme activities, gene expression levels and WSC levels, suggests that phytohormones do apparently not play a major role in the short term regulation of fructan metabolism. However, contrary to what happens at a response to cold inducing fructan accumulation parallel to the increase of sucrose level (Hisano et al., 2008), the sucrose production rate may be too low to allow fructan accumulation. Further, we cannot exclude that differences between independent experiments originate from different uptake of the phytohormone into the plant tissue. In addition, the variability between the different experiments (Supplemental Figure 1, Supplemental Tables 1–10) indicates that also other external factors beyond our control and internal regulatory mechanisms also affect the studied enzymatic activities and transcriptional regulation.

Based on the effect of the five tested phytohormones on the expression and activities of some enzymes involved in fructan metabolism, as well as key enzymes involved in primary carbohydrate metabolism, the phytohormones can be divided in three groups. The first group consists of ABA and ET, representing classical stress phytohormones, among which especially ABA is involved in abiotic stress responses (Nakashima et al., 2009) and remobilization of fructans by FEH (Yang et al., 2004). The second group consists of AUX and KIN, involved in plant growth and development. An increasing number of reports provide evidence that auxins and cytokinins also function during stress responses (Shibasaki et al., 2009; Suárez-González et al., 2014). Finally, GA represents a growth phytohormone, which is often affected by stress responses ultimately causing altered/reduced growth through GA signaling. Rigui et al. (2015) have shown that ABA and AUX levels correlated with fructan metabolism in *Chrysolaena obovate* and Valluru (2015) has proposed a model for the function of ABA, AUX and ET in the regulation of fructan biosynthesis and breakdown. GA treatment caused a reduction in both *FEH* expression and 1-FEH activity. In contrast, Morvan et al. (1997) reported in a previous study that continuous treatment of defoliated perennial ryegrass plants with GA_3_ (10 μM) in the nutrient solution in combination with a spraying pulse of uniconazole (an inhibitor of GA biosynthesis) maintained FEH activity at control level, while spraying of uniconazole alone caused a decrease in FEH activity compared to the control at 24 h post uniconazole treatment. Interestingly, the combination of uniconazole and 10 μM GA resulted in higher FEH activity at 48 h post uniconazole treatment compared to the control. Reduced FEH activity in uniconazole treated plants might be due to its ability to repress GA synthesis and/or to increase the endogenous ABA levels. Absence of 1-FEH activity modulation by GA and ABA in the present study suggests that the other FEH isoforms of *L. perenne* (Lothier et al., 2007, 2014) might be differently regulated by these phytohormones.

An additional important novel aspect of our study is that we assessed the link between fructan and primary carbohydrate metabolism by investigating the activities for key enzymes regulating the sucrose pool (Jammer et al., 2015), the substrate for fructan synthesis. FTs are supposed to originate from acidic vacuolar invertases (Vijn and Smeekens, 1999; Wei and Chatterton, 2001) and FEHs from cell wall invertases (Le Roy et al., 2007). Thus, the enzymes involved in fructan metabolism might share regulatory mechanisms with these acidic invertases. Since vacuolar invertase and FTs would compete for sucrose as substrate and invertases were reported to degrade lowDP fructans (Livingston et al., 2009), differential regulation should occur to enable fructan accumulation. However, FT and vacuolar invertase activity was initially induced by all phytohormones (except KIN) in parallel under our experimental conditions, suggesting that indeed regulatory mechanisms are shared between FTs and vacuolar invertase. In contrast, in wheat high fructan levels were correlated with high *FT* expression levels, but inversely correlated with both invertase expression and enzyme activities (Xue et al., 2008). Therefore, long term studies in perennial ryegrass are needed to understand the link between fructan and primary carbohydrate metabolism, specifically on the possible competition for sucrose by FTs and vacuolar invertase. Only for 1-FEH and cell wall invertase activity different responses upon phytohormone treatment were observed in our study. While 1-FEH activity was increased by KIN, cell wall invertase activity was not significantly altered. Effects of ABA, ET, and GA treatment on cell wall invertase activity were inconsistent and therefore difficult to interpret.

Hexoses are released during fructan accumulation (glucose) or degradation (fructose). Therefore, other enzymes regulating the sucrose pool and, thereby, futile cycling of sucrose could also be important for understanding the regulation of fructan metabolism. UGPase activity is crucial for sucrose biosynthesis in catalyzing the intermediate step from glucose-1-phosphate to UDP-glucose (Halford et al., 2011). Increased UGPase activity could provide UDP-glucose to maintain a futile cycle of sucrose. The cytosolic isoform of G6PDH, which represents 78–92% of the total activity (Cardi et al., 2011), competes for G6P with cytosolic PGM, involved in sucrose synthesis. Sarkar et al. (2009) reported an increase of G6PDH activity in cold acclimated perennial ryegrass, independent from phenolic compounds and antioxidant mechanisms. The protective potential of NADPH derived from G6PDH toward stress conditions in animal cells, via NADPH oxidases, lipid synthesis enzymes and antioxidant enzymes, was recently reviewed by Stanton (2012). Thus, G6PDH activity might feed NADPH into the membrane stabilization processes under hardening or stress conditions, while a reduced activity of G6PDH would allow accumulation of G6P, which in turn is known to induce sucrose biosynthesis through activation of sucrose phosphate synthase (SPS) (regulation of sucrose metabolism reviewed by Ruan, 2014).

Although often a poor correlation between mRNA levels and enzyme activities and/or protein levels are found, a good correlation between *SST* expression and 1-SST enzyme activity was reported for perennial ryegrass leaf sheaths and elongating leaf bases under fructan accumulating conditions (Lasseur et al., 2006). In our study, only a good correlation between the gene expression levels and enzyme activities were evident for 1-SST (ET) and 6G-FFT (GA), while for 1-SST (AUX, KIN) and 6G-FFT (ET) weak correlations were observed. Further, given that the weak changes in *1-FEH* expression were not significant, the *1-FEH* expression levels would correlate with the 1-FEH enzyme activities for ET, AUX and GA. FTs in sink tissue of perennial ryegrass seem to be predominantly regulated at the transcriptional level (Prud'homme et al., 2007). However, Lasseur et al. (2006) found higher *6G-FFT* and *SST* mRNA levels in leaf sheaths, but higher 6G-FFT and 1-SST activity in elongating leaf bases, thus suggesting a tissue specific regulation. Because of high protein stability, weak transcriptional changes can result in cumulative higher protein levels in time and thus enzyme activities, while enzyme activities can mask fast changes or fluctuations in gene expression levels. Recently, Hisano et al. (2008) revealed that several genes encode isozymes for the same FT activity in perennial ryegrass. Lothier et al. (2014) suggested FEH isozymes that would preferentially degrade either low or highDP fructans, based on findings on substrate specificity of FEHs from perennial ryegrass (Marx et al., 1997; Lothier et al., 2007, 2014) and wheat (Kawakami et al., 2005). Thus, expression of a single gene does not have to be correlated with actual enzyme activity in extracts, especially when the FT or FEH activity results from several isozymes. In a long-term study on *in vitro* cultured *Agave tequilana* plantlets, Suárez-González et al. (2014) recently reported an accumulation of lowDP fructans (DP 3–5) in stems 24 days after treatment with 10 or 50 μM ABA, 1mg.l^−1^ KIN, 1 mM salicylic acid, 50 μM methyl jasmonate, 8% sucrose, and cold treatment (10 d, 0°C). Total fructan content was highest in stems treated with sucrose, 50 μM ABA and 1 mM salicylic acid 42 days after treatment. Elevated fructan content was accompanied by higher *1-SST* and *1-FFT* transcript levels 24 and 42 d post 50 μM ABA, 1mg.l^−1^ KIN or sucrose treatment. Long-term treatment (2 months) with concentration of AUX (13.4 μM) similar to the one used in our present study led to an increase in total fructan of *in vitro* grown agave (Barreto et al., 2010). Our findings that pulse treatments with ABA, KIN, and AUX caused (transient) increases in FT activities are in agreement with these studies. The lack of clear changes in WSC levels in our study are most likely caused by our short time experiments, indicating the importance to study enzyme activities as integrator of transcriptional and post-translational regulations to obtain insights in regulatory mechanisms from a shorter experimental time span than derived from WSC levels. Thus, enzyme activities could potentially be predictive and robust markers and thus used as proxy for only later observable traits such as a high fructan content for practical applications in breeding. It is beeing recognized that the plant physiology is a central integrator of both biotic and abiotic environmental factors and, for crop plants, agricultural management, and that the enzymes activities are more closely related to the phenotypic appearance than transcript levels (Großkinsky et al., 2015).

To study the possible link between fructan and primary carbohydrate metabolism at the level of enzyme activities is a novel aspect within a physiological phenotyping approach (Großkinsky et al., 2015) compared to previous studies and very important since (1) FTs and FEHs are supposed to originate from acidic invertases and thus might share similar regulatory mechanisms, (2) FTs and vacuolar invertase use sucrose as substrate and thus might compete for the same available substrate, and (3) invertases were reported to degraded low DP fructans. Therefore, simultaneous induction of FT, FEH, and acidic invertase activities could strongly limit fructan accumulation, while the futile cycling of sucrose might be crucial for the regulation of fructan metabolism, enabling efficient use of the hexoses that are released during fructan accumulation (glucose) or degradation (fructose). This is especially important in case of parallel induction of FT and FEH activities. However, the present study does not support a fast co-regulation by the tested five phytohormones under the experimental conditions used.

While exogenous application of phytohormones is widely used to assess their function in non-model plant species that are not readily assessable for functional approaches with transgenic plants, this approach has also certain limitations to draw general conclusions about the role of the phytohormone tested. Thus, alternative approaches to reduce the level of biological active phytohormones via the use of inhibitors to interfere with their synthesis or via accelerating their degradation, or the use of antagonists of their biological activity need to be considered, although only a limited number of such compounds are available and many of them have also a limited specificity.

Although we have generally observed only subtle effects with the selected single concentrations applied, it is not expected that significantly lower or higher concentrations will have a stronger physiological effect. Significantly higher concentrations of phytohormones (50–100 μM) have to be considered as non-physiological and would very likely not reflect the natural physiological situation in plants. The use of unphysiologically high concentrations generally bears also the danger that the dominating effect is due to the chemical nature of the phytohormone tested rather than the physiological phytohormone effect, which is in particular problematic in the case of phytohormones with acidic chemical nature. A recently published study shows that the response of ryegrass to exogenously applied GAs is strongly influenced by developmental triggers and also various abiotic factors (Parsons et al., 2013). Thus, GAs may have under certain conditions or developmental stages only very limited effects on ryegrass, which is in agreement with our study. Nevertheless, our broad and comprehensive approach studying the impact of five phytohormones using only a single, commonly used concentration for each phytohormone has the inherent limitation that possible small optimal concentration ranges were missed. However, our study provides a starting point for further analysis of hormonal effects for the five classical phytohormones using detailed concentration ranges for the individual single phytohormones. Thus, our manuscript provides an expansion of the knowledge on the short term effect of phyohormones on the regulation of fructan metabolism and the possible link with primary carbohydrate metabolism in perennial ryegrass.

## Author contributions

Designed the experiments: AG, AM, MP, EV, and TR. Performed the experiments: AG. Analyzed the data: AG, AM, and EV. Contributed reagents/materials/analysis tools: MP, EV, and TR. Wrote the paper: AG, AM, MP, EV, and TR.

### Conflict of interest statement

The authors declare that the research was conducted in the absence of any commercial or financial relationships that could be construed as a potential conflict of interest.

## Abbreviations

1-FFT: fructan:fructan 1-fructosyltransferase
1-SST: sucrose:sucrose 1-fructosyltransferase
6G-FFT: fructan:fructan 6G-fructosyltransferase, 6PG, 6-phospho gluconate
6-SFT: sucrose:fructan 6-fructosyltransferase
1-FEH: fructan 1-exohydrolase, 6-FEH, fructan 6-exohydrolase
ABA: abscisic acid, Ac Co A, acetyl coenzyme A
ADP: adenosine triphosphate, ADP-Gluc: adenosine triphosphate glucose, AGPase: adenosine diphosphate glucose pyrophosphorylase, ATP: adenosine triphosphate, AUX, auxin
cwInv: cell wall bound invertase
cytInv: cytosolic invertase
DHAP: dihydroxyacetone phosphate
DP: polymerization degree
*eEF-1*α: *elongation factor 1*-α, ET, ethephon
F1,6bP: fructose-1,6-bisphosphate
F6P: fructose-6-phosphate
FbP-ALD: fructose-1,6-bisphosphate dependent aldolase
FK: fructokinase
Fru: fructose
FT: fructosyltransferase
G1P: glucose-1-phosphate
G3P: 3-phospho glycerate, G6P, glucose-6-phosphate
G6PT: glucose-6-phosphat translocator
G6PDH: glucose-6-phosphate dehydrogenase
GA: gibberellic acid
*GAPDH*: *glyceraldehyde 3-phosphate dehydrogenase*
Glu: glucose
HK: hexokinase
HPLC: high performance liquid chromatography
KIN: kinetin
*Lp*: *Lolium perenne*
NAD(P)+: nicotinamid adenine dinucleotide (phosphate)(_ox_)
NAD(P)H: nicotinamid adenine dinucleotide (phosphate)(_red_)
PFK: phosphofructokinase
PGI: phosphoglucose isomerase
PGM: phosphoglucose mutase
P*i*/PP*i*: inorganic phosphate
PTP: pyruvate transport protein
PVPP: polyvinylpyrrolidone
SPP: sucrose phosphate phosphatase
SPS: sucrose phosphate synthase
Suc: sucrose
SuSy: sucrose synthase
SUT: sucrose transporter
TPI: triosephosphate isomerase
TPT: triose-phosphate/phosphate translocator
UDP: uracyl diphosphate
UDP-Glu: uridinyl diphosphate glucose
UGPase: uridine 5'-diphosphoclucose pyrophosphorylase
UTP: uridine triphosphate
vacInv: vacuolar invertase
WSC: water soluble carbohydrates.
