# Supplementary material for: Exogenous Classic Phytohormones Have Limited Regulatory Effects on Fructan and Primary Carbohydrate Metabolism in Perennial Ryegrass (Lolium perenne L.)
Source: Front Plant Sci. 2016 Jan 20;6:1251. doi: 10.3389/fpls.2015.01251 (PMC4719101; doi:10.3389/fpls.2015.01251)
Supplement: Supplementary file 1 [file DataSheet1.pdf]

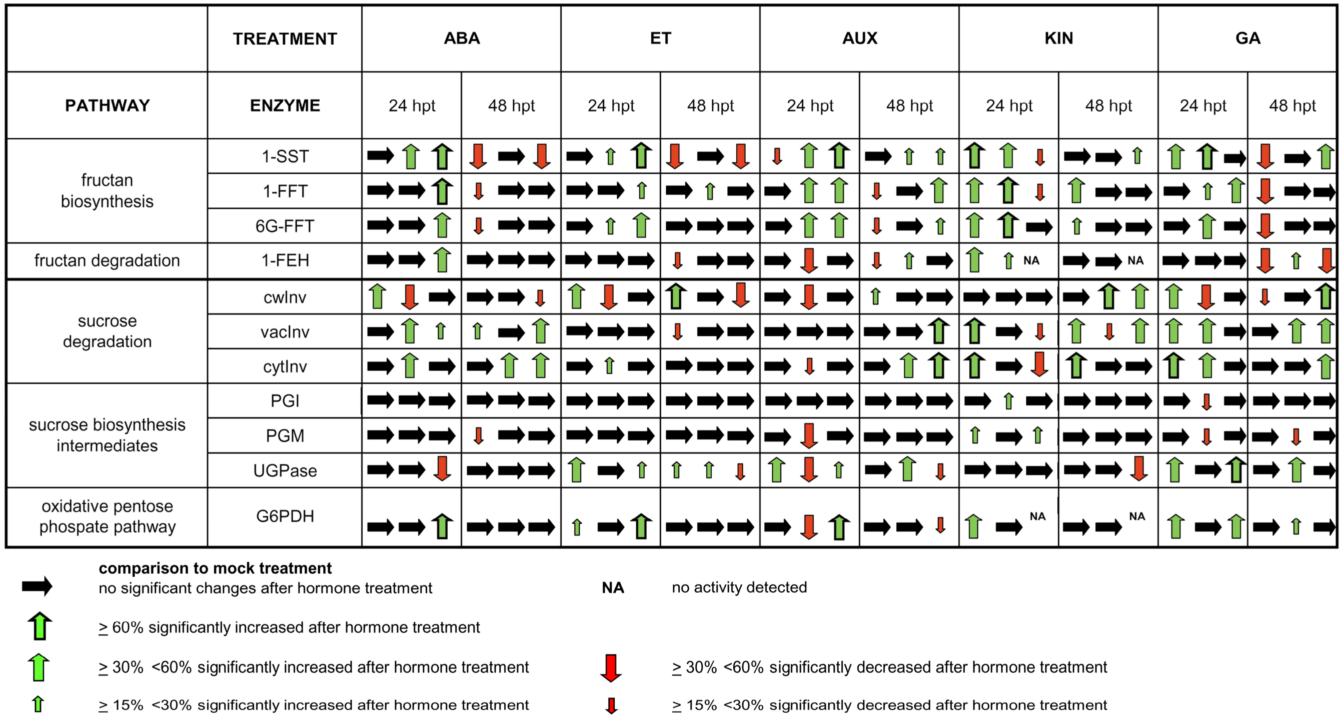

Supplemental Figure 1. Overview of fructan and carbohydrate enzyme activities in the three independent hormone treatment experiments. Changes in cell wall bound invertase (cwInv), vacuolar invertase (vacInv), cytoplasmic invertase (cytInv), phosphoglucose isomerase (PGI), phosphoglucose mutase (PGM), UDP-glucose pyrophosphorylase (UGPase), glucose 6-phosphat dehydrogenase (G6PDH), 1-sucrose:sucrose fructosyltransferase (1-SST), fructan:fructan 6G-fructosyltransferase (6G-FFT), fructan:fructan 1-fructosylstransferase (1-FFT) and fructan-1-exohydrolase (1-FEH) activities in stubble of 4-wk-old *Lolium perenne* Aberchoice plants treated with either 10 µM abscisic acid (ABA), 0.15% ethephon (ET), 10 µM auxin (AUX), 10 µM kinetin (KIN) or 10 µM gibberellic acid (GA) compared to the corresponding mock treatments.

Supplemental Table 1. Cell wall bound invertase (cwInv), vacuolar invertase (vacInv), cytoplasmic invertase (cytInv), phosphoglucose isomerase (PGI), phosphoglucose mutase (PGM), UDP-glucose pyrophosphorylase (UGPase), glucose 6-phosphat dehydrogenase (G6PDH), 1-sucrose:sucrose fructosyltransferase (1-SST), fructan:fructan 6G-fructosyltransferase (6G-FFT), fructan:fructan 1-fructosylstransferase (1-FFT) and fructan-1-exohydrolase (1-FEH) activities [nkat.gFW^{-1}] in stubble of 4-wk-old *Lolium perenne* Aberchoice plants harvested prior to treatment (Control), and 24 (24 hpt) or 48 hours (48 hpt) post treatment with water (Mock) or 10 µM abscisic acid. Enzymatic activities were analyzed in the first centimeter of stubble from ground level. Values in experiment 1 represent the mean of two, in experiments 2 and 3 the mean of three biological samples ± SE. Asterisks indicate values of enzymatic activities statistically different from mock treatment (analyzed by Student's t-test). * P < 0.05, ** P < 0.01, *** P < 0.001. Percent change in enzymatic activities between mock and abscisic acid treatment is given as Δ [%].

Enzyme	Specific activity [nkat.gFW^{-1}] Experiment 1			Δ [%]	Specific activity [nkat.gFW^{-1}] Experiment 2			Δ [%]	Specific activity [nkat.gFW^{-1}] Experiment 3			Δ [%]
	Control	Mock	Abscisic acid		Control	Mock	Abscisic acid		Control	Mock	Abscisic acid	
	0 hpt	24 hpt	24 hpt		0 hpt	24 hpt	24 hpt		0 hpt	24 hpt	24 hpt	
cwInv	0.61 ± 0.07	0.36 ± 0.02	0.46 ± 0.06 *	31	1.10 ± 0.11	0.80 ± 0.11	0.43 ± 0.03 **	-46	0.46 ± 0.02	0.65 ± 0.07	0.66 ± 0.05	6
vacInv	0.84 ± 0.11	0.62 ± 0.02	0.64 ± 0.05	3	0.59 ± 0.03	0.49 ± 0.02	0.67 ± 0.03 ***	37	0.88 ± 0.04	0.63 ± 0.07	0.85 ± 0.08 *	25
cytInv	0.30 ± 0.01	0.20 ± 0.04	0.26 ± 0.02	26	0.28 ± 0.01	0.21 ± 0.01	0.28 ± 0.03 *	33	0.55 ± 0.03	0.50 ± 0.04	0.59 ± 0.06	18
PGI	23.64 ± 2.74	24.28 ± 0.37	21.47 ± 3.18	-12	15.44 ± 0.44	18.69 ± 0.43	18.42 ± 0.25	-1	8.06 ± 0.19	7.89 ± 0.44	8.70 ± 0.19	10
PGM	45.81 ± 3.65	37.75 ± 0.72	38.89 ± 1.59 **	3	39.37 ± 1.39	40.85 ± 0.67	34.90 ± 2.95	-15	20.35 ± 0.22	17.20 ± 1.78	21.03 ± 1.05	22
UGPase	65.78 ± 12.06	68.32 ± 1.17	69.40 ± 2.96 ***	2	63.17 ± 5.90	66.04 ± 10.85	60.00 ± 11.47	-9	37.31 ± 0.97	37.81 ± 1.75	21.91 ± 2.29 ***	-42
G6PDH	2.96 ± 0.84	5.30 ± 0.54	5.34 ± 0.43	1	5.19 ± 0.23	5.15 ± 0.21	4.71 ± 0.48	-8	3.28 ± 0.19	2.27 ± 0.33	3.65 ± 0.39 *	61
1-SST	1.51 ± 0.03	2.04 ± 0.08	1.75 ± 0.13 *	-14	0.46 ± 0.07	0.41 ± 0.01	0.57 ± 0.05 **	37	0.25 ± 0.02	0.22 ± 0.01	0.53 ± 0.05 ***	143
6G-FFT	7.41 ± 0.36	7.35 ± 0.62	7.29 ± 0.18	-1	4.16 ± 0.23	5.01 ± 0.15	5.74 ± 0.41	14	2.62 ± 0.10	2.79 ± 0.17	4.44 ± 0.17 ***	59
1-FFT	2.78 ± 0.37	2.45 ± 0.08	2.42 ± 0.16	-1	0.52 ± 0.07	0.81 ± 0.03	0.85 ± 0.03	5	0.74 ± 0.03	0.81 ± 0.06	1.30 ± 0.11 ***	61
1-FEH	0.22 ± 0.01	0.20 ± 0.01	0.29 ± 0.04	42	0.15 ± 0.01	0.14 ± 0.01	0.15 ± 0.01	9	0.13 ± 0.01	0.12 ± 0.01	0.16 ± 0.01 ***	32
	0 hpt	48 hpt	48 hpt		0 hpt	48 hpt	48 hpt		0 hpt	48 hpt	48 hpt	
cwInv	0.61 ± 0.07	0.53 ± 0.01	0.70 ± 0.10	31	1.10 ± 0.11	0.80 ± 0.07	0.40 ± 0.03	-25	0.46 ± 0.02	0.88 ± 0.06	0.68 ± 0.05 *	-23
vacInv	0.84 ± 0.11	0.63 ± 0.02	0.80 ± 0.16 **	28	0.59 ± 0.03	0.48 ± 0.02	0.45 ± 0.01	-5	0.88 ± 0.04	0.66 ± 0.04	0.93 ± 0.01 ***	42
cytInv	0.30 ± 0.01	0.23 ± 0.02	0.25 ± 0.01	6	0.28 ± 0.01	0.46 ± 0.03	0.65 ± 0.05 **	40	0.55 ± 0.03	0.33 ± 0.04	0.52 ± 0.02 ***	56
PGI	23.64 ± 2.74	25.39 ± 0.62	22.07 ± 0.44	-13	15.44 ± 0.44	21.48 ± 0.42	21.59 ± 0.56	1	8.06 ± 0.19	7.32 ± 0.22	7.59 ± 0.10	4
PGM	45.81 ± 3.65	47.78 ± 3.04	34.41 ± 0.90 **	-28	39.37 ± 1.39	45.74 ± 0.77	45.84 ± 1.53	0	20.35 ± 0.22	22.02 ± 0.21	21.43 ± 0.44	-3
UGPase	65.78 ± 12.06	80.45 ± 0.64	85.82 ± 9.95 *	7	63.17 ± 5.90	55.42 ± 4.05	65.01 ± 5.15	17	37.31 ± 0.97	54.96 ± 2.08	52.57 ± 2.04	-4
G6PDH	2.96 ± 0.84	5.79 ± 0.54	6.32 ± 0.31	9	5.19 ± 0.23	4.51 ± 0.17	5.04 ± 0.36	12	3.28 ± 0.19	2.95 ± 0.22	2.67 ± 0.28	-10
1-SST	1.51 ± 0.03	2.47 ± 0.22	1.65 ± 0.09 **	-33	0.46 ± 0.07	0.54 ± 0.04	0.45 ± 0.09	-16	0.25 ± 0.02	0.36 ± 0.03	0.25 ± 0.02 **	-31
6G-FFT	7.41 ± 0.36	10.85 ± 0.98	8.53 ± 0.46 *	-21	4.16 ± 0.23	7.51 ± 0.96	8.36 ± 0.40	11	2.62 ± 0.10	3.14 ± 0.08	3.24 ± 0.07	3
1-FFT	2.78 ± 0.37	3.42 ± 0.25	2.65 ± 0.10 **	-23	0.52 ± 0.07	1.05 ± 0.06	1.21 ± 0.23	15	0.74 ± 0.03	0.71 ± 0.04	0.80 ± 0.03	12
1-FEH	0.22 ± 0.01	0.18 ± 0.01	0.17 ± 0.00	-8	0.15 ± 0.01	0.15 ± 0.00	0.16 ± 0.010 *	10	0.13 ± 0.01	0.10 ± 0.01	0.14 ± 0.02	38

Supplemental Table 2. Cell wall bound invertase (cwInv), vacuolar invertase (vacInv), cytoplasmic invertase (cytInv), phosphoglucose isomerase (PGI), phosphoglucose mutase (PGM), UDP-glucose pyrophosphorylase (UGPase), glucose 6-phosphat dehydrogenase (G6PDH), 1-sucrose:sucrose fructosyltransferase (1-SST), fructan:fructan 6G-fructosyltransferase (6G-FFT), fructan:fructan 1-fructosylstransferase (1-FFT) and fructan-1-exohydrolase (1-FEH) activities [nkat.gFW^{-1}] in stubble of 4-wk-old *Lolium perenne* Aberchoice plants harvested prior to treatment (Control), and 24 (24 hpt) or 48 hours (48 hpt) post treatment with water (Mock) or 0.15% ethephon. Enzymatic activities were analyzed in the first centimeter of stubble from ground level. Values in experiment 1 represent the mean of two, in experiments 2 and 3 the mean of three biological samples ± SE. Asterisks indicate values of enzymatic activities statistically different from mock treatment (analyzed by Student's t-test). * P < 0.05, ** P < 0.01, *** P < 0.001. Percent change in enzymatic activities between mock and ethephon treatment is given as Δ [%].

Enzyme	Specific activity [nkat.gFW^{-1}] Experiment 1			Δ [%]	Specific activity [nkat.gFW^{-1}] Experiment 2			Δ [%]	Specific activity [nkat.gFW^{-1}] Experiment 3			Δ [%]
	Control	Mock	Ethephon		Control	Mock	Ethephon		Control	Mock	Ethephon	
	0 hpt	24 hpt	24 hpt		0 hpt	24 hpt	24 hpt		0 hpt	24 hpt	24 hpt	
cwInv	0.61 ± 0.07	0.36 ± 0.02	0.47 ± 0.03 **	31	1.10 ± 0.11	0.80 ± 0.11	0.46 ± 0.04 **	-42	0.46 ± 0.02	0.65 ± 0.07	0.68 ± 0.02	9
vacInv	0.84 ± 0.11	0.63 ± 0.02	0.54 ± 0.01 **	-14	0.59 ± 0.04	0.49 ± 0.02	0.55 ± 0.01 *	13	0.88 ± 0.04	0.63 ± 0.07	0.74 ± 0.02	9
cytInv	0.30 ± 0.01	0.20 ± 0.04	0.23 ± 0.02	14	0.28 ± 0.01	0.21 ± 0.01	0.25 ± 0.01 ***	21	0.55 ± 0.03	0.51 ± 0.04	0.51 ± 0.02	2
PGI	23.64 ± 2.74	24.28 ± 0.37	24.57 ± 0.87	1	15.44 ± 0.44	18.69 ± 0.43	19.34 ± 0.77	3	8.06 ± 0.19	7.89 ± 0.44	7.40 ± 0.29	-6
PGM	45.81 ± 3.64	37.75 ± 0.73	44.36 ± 3.55	18	39.37 ± 1.39	40.85 ± 0.67	39.75 ± 1.29	-3	20.35 ± 0.22	17.20 ± 1.78	20.77 ± 0.68	21
UGPase	65.78 ± 12.06	68.32 ± 1.17	96.73 ± 5.11 **	42	63.17 ± 5.90	66.04 ± 10.85	65.32 ± 6.33	-1	37.31 ± 0.97	37.81 ± 1.75	44.71 ± 0.78 **	18
G6PDH	2.96 ± 0.84	5.30 ± 0.53	6.58 ± 0.19 *	24	5.19 ± 0.23	5.15 ± 0.22	5.68 ± 0.13 *	10	3.28 ± 0.19	2.27 ± 0.33	3.66 ± 0.28 **	61
1-SST	1.51 ± 0.03	2.04 ± 0.08	2.45 ± 0.65	20	0.46 ± 0.07	0.41 ± 0.01	0.52 ± 0.01 **	26	0.25 ± 0.02	0.22 ± 0.01	0.37 ± 0.03 **	70
6G-FFT	7.41 ± 0.36	7.35 ± 0.62	7.07 ± 0.23	-4	4.16 ± 0.23	5.01 ± 0.15	6.23 ± 0.19 ***	24	2.62 ± 0.10	2.79 ± 0.17	3.76 ± 0.12 ***	35
1-FFT	2.78 ± 0.37	2.45 ± 0.08	2.16 ± 0.06 **	-12	0.52 ± 0.07	0.81 ± 0.03	0.88 ± 0.08	8	0.74 ± 0.03	0.81 ± 0.06	1.00 ± 0.03 *	24
1-FEH	0.22 ± 0.01	0.20 ± 0.01	0.18 ± 0.01	-10	0.15 ± 0.01	0.14 ± 0.01	0.16 ± 0.00 *	14	0.13 ± 0.01	0.12 ± 0.01	0.14 ± 0.03	14
	0 hpt	48 hpt	48 hpt		0 hpt	48 hpt	48 hpt		0 hpt	48 hpt	48 hpt	
cwInv	0.61 ± 0.07	0.53 ± 0.05	0.92 ± 0.28 *	72	1.10 ± 0.11	0.53 ± 0.07	0.48 ± 0.01	-11	0.46 ± 0.02	0.88 ± 0.06	0.49 ± 0.02 ***	-46
vacInv	0.84 ± 0.11	0.63 ± 0.02	0.47 ± 0.05 *	-24	0.59 ± 0.04	0.48 ± 0.02	0.45 ± 0.03	0	0.88 ± 0.04	0.66 ± 0.04	0.66 ± 0.02	-1
cytInv	0.30 ± 0.01	0.23 ± 0.02	0.23 ± 0.03	-4	0.28 ± 0.01	0.46 ± 0.03	0.55 ± 0.04	18	0.55 ± 0.03	0.33 ± 0.04	0.32 ± 0.01	-4
PGI	23.64 ± 2.74	25.39 ± 0.62	24.67 ± 1.08	-3	15.44 ± 0.44	21.48 ± 0.42	22.01 ± 0.24	2	8.06 ± 0.19	7.32 ± 0.22	7.12 ± 0.09	3
PGM	45.81 ± 3.64	47.78 ± 3.04	48.62 ± 6.92	2	39.37 ± 1.39	45.74 ± 0.78	43.38 ± 0.69 *	-5	20.35 ± 0.22	22.02 ± 0.21	19.42 ± 0.68 **	-13
UGPase	65.78 ± 12.06	80.45 ± 0.64	97.60 ± 1.73 ***	21	63.17 ± 5.90	55.42 ± 4.05	68.46 ± 2.82 **	24	37.31 ± 0.97	54.96 ± 2.08	45.89 ± 2.07 **	-17
G6PDH	2.96 ± 0.84	5.79 ± 0.55	6.78 ± 1.26	17	5.19 ± 0.23	4.51 ± 0.17	5.06 ± 0.12 **	12	3.28 ± 0.19	2.95 ± 0.22	3.16 ± 0.12	7
1-SST	1.51 ± 0.03	2.47 ± 0.22	1.64 ± 0.09 **	-34	0.46 ± 0.07	0.54 ± 0.04	0.46 ± 0.06	-14	0.25 ± 0.02	0.36 ± 0.03	0.16 ± 0.01 ***	-56
6G-FFT	7.41 ± 0.36	10.85 ± 0.98	9.17 ± 0.10	-15	4.16 ± 0.23	7.51 ± 0.96	8.51 ± 0.22	13	2.62 ± 0.10	3.14 ± 0.08	3.15 ± 0.06	0
1-FFT	2.78 ± 0.37	3.42 ± 0.25	3.13 ± 0-60	-8	0.52 ± 0.07	1.05 ± 0.06	1.29 ± 0.06 **	23	0.74 ± 0.03	0.71 ± 0.04	0.76 ± 0.02	6
1-FEH	0.22 ± 0.01	0.18 ± 0.01	0.15 ± 0.00 *	-18	0.15 ± 0.01	0.15 ± 0.00	0.15 ± 0.01	0	0.13 ± 0.01	0.10 ± 0.01	0.12 ± 0.01	19

Supplemental Table 3. Cell wall bound invertase (cwInv), vacuolar invertase (vacInv), cytoplasmic invertase (cytInv), phosphoglucose isomerase (PGI), phosphoglucose mutase (PGM), UDP-glucose pyrophosphorylase (UGPase), glucose 6-phosphat dehydrogenase (G6PDH), 1-sucrose:sucrose fructosyltransferase (1-SST), fructan:fructan 6G-fructosyltransferase (6G-FFT), fructan:fructan 1-fructosylstransferase (1-FFT) and fructan-1-exohydrolase (1-FEH) activities [nkat.gFW^{-1}] in stubble of 4-wk-old *Lolium perenne* Aberchoice plants harvested prior to treatment (Control), and 24 (24 hpt) or 48 hours (48 hpt) post treatment with water (Mock) or 10 µM auxin. Enzymatic activities were analyzed in the first centimeter of stubble from ground level. Values in experiment 1 represent the mean of two, in experiments 2 and 3 the mean of three biological samples ± SE. Asterisks indicate values of enzymatic activities statistically different from mock treatment (analyzed by Student's t-test). * $P < 0.05$, ** $P < 0.01$, *** $P < 0.001$. Percent change in enzymatic activities between mock and auxin treatment is given as Δ [%].

Enzyme	Specific activity [nkat.gFW^{-1}] Experiment 1			Δ [%]	Specific activity [nkat.gFW^{-1}] Experiment 2			Δ [%]	Specific activity [nkat.gFW^{-1}] Experiment 3			Δ [%]
	Control	Mock	Auxin		Control	Mock	Auxin		Control	Mock	Auxin	
	0 hpt	24 hpt	24 hpt		0 hpt	24 hpt	24 hpt		0 hpt	24 hpt	24 hpt	
cwInv	0.61 ± 0.07	0.36 ± 0.02	0.35 ± 0.01	-2	1.10 ± 0.11	0.80 ± 0.11	0.37 ± 0.04 **	-53	0.46 ± 0.02	0.62 ± 0.07	0.75 ± 0.05	20
vacInv	0.84 ± 0.11	0.62 ± 0.02	0.65 ± 0.06	4	0.59 ± 0.03	0.49 ± 0.02	0.48 ± 0.03	-3	0.88 ± 0.04	0.63 ± 0.07	0.73 ± 0.03	8
cytInv	0.30 ± 0.01	0.20 ± 0.04	0.24 ± 0.01	17	0.28 ± 0.01	0.21 ± 0.01	0.15 ± 0.01 **	-27	0.55 ± 0.03	0.51 ± 0.04	0.44 ± 0.03	-13
PGI	23.64 ± 2.74	24.28 ± 0.37	24.02 ± 1.34	-1	15.44 ± 0.44	18.69 ± 0.43	17.93 ± 0.67	-4	8.06 ± 0.19	7.89 ± 0.44	7.84 ± 0.28	-1
PGM	45.81 ± 3.64	37.75 ± 0.73	37.06 ± 4.66	-2	39.37 ± 1.39	40.85 ± 0.67	22.52 ± 2.78 ***	-45	20.35 ± 0.22	17.20 ± 1.78	20.22 ± 0.71	18
UGPase	65.78 ± 12.06	68.32 ± 1.17	93.66 ± 2.54 ***	37	63.17 ± 5.90	66.04 ± 10.85	26.86 ± 1.79 **	-59	37.31 ± 0.97	37.81 ± 1.75	45.62 ± 3.24 *	21
G6PDH	2.96 ± 0.84	5.30 ± 0.53	4.64 ± 0.20	-12	5.19 ± 0.23	5.15 ± 0.22	2.85 ± 0.13 **	-45	3.28 ± 0.19	2.27 ± 0.33	3.81 ± 0.38 **	68
1-SST	1.51 ± 0.03	2.04 ± 0.08	1.56 ± 0.09 ***	-24	0.46 ± 0.07	0.41 ± 0.01	0.66 ± 0.03 ***	59	0.25 ± 0.02	0.22 ± 0.01	0.47 ± 0.06 ***	116
6G-FFT	7.41 ± 0.51	7.35 ± 0.62	7.62 ± 0.53	4	4.16 ± 0.46	5.01 ± 0.15	7.42 ± 0.17 ***	48	2.62 ± 0.19	2.79 ± 0.17	4.30 ± 0.26 ***	54
1-FFT	2.78 ± 0.37	2.45 ± 0.08	2.80 ± 0.12 *	14	0.52 ± 0.07	0.81 ± 0.03	1.14 ± 0.05 ***	41	0.74 ± 0.03	0.81 ± 0.06	1.24 ± 0.09 ***	54
1-FEH	0.22 ± 0.01	0.20 ± 0.01	0.21 ± 0.01	2	0.15 ± 0.01	0.14 ± 0.01	0.09 ± 0.01 *	-31	0.13 ± 0.01	0.12 ± 0.01	0.16 ± 0.01 **	29
	0 hpt	48 hpt	48 hpt		0 hpt	48 hpt	48 hpt		0 hpt	48 hpt	48 hpt	
cwInv	0.61 ± 0.07	0.53 ± 0.05	0.69 ± 0.01 ***	29	1.10 ± 0.11	0.53 ± 0.07	0.50 ± 0.04	-7	0.46 ± 0.02	0.88 ± 0.06	1.08 ± 0.08	22
vacInv	0.84 ± 0.11	0.63 ± 0.02	0.69 ± 0.01 *	10	0.59 ± 0.03	0.48 ± 0.02	0.50 ± 0.04	3	0.88 ± 0.04	0.66 ± 0.04	1.08 ± 0.08 ***	64
cytInv	0.30 ± 0.01	0.23 ± 0.02	0.25 ± 0.03	8	0.28 ± 0.01	0.46 ± 0.03	0.59 ± 0.03 **	29	0.55 ± 0.03	0.33 ± 0.04	0.64 ± 0.06 ***	92
PGI	23.64 ± 2.74	25.39 ± 0.62	23.88 ± 1.51	-6	15.44 ± 0.44	21.48 ± 0.84	19.00 ± 0.28 ***	-12	8.06 ± 0.19	7.32 ± 0.22	7.24 ± 0.25	-1
PGM	45.81 ± 3.64	47.78 ± 3.04	37.06 ± 4.66	-22	39.37 ± 1.39	45.74 ± 0.77	40.25 ± 1.22 ***	-12	20.35 ± 0.22	22.02 ± 0.21	20.73 ± 0.39 **	-6
UGPase	65.78 ± 12.06	80.45 ± 0.64	59.02 ± 11.24	-26	63.17 ± 5.90	55.42 ± 4.05	76.98 ± 5.41 **	39	37.31 ± 0.97	54.96 ± 2.08	44.08 ± 0.79 ***	-20
G6PDH	2.96 ± 0.84	5.79 ± 0.55	4.64 ± 0.20	-20	5.19 ± 0.23	4.51 ± 0.17	5.05 ± 0.27	12	3.28 ± 0.19	2.95 ± 0.22	2.32 ± 0.17 *	-21
1-SST	1.51 ± 0.03	2.47 ± 0.22	2.12 ± 0.21	-14	0.46 ± 0.07	0.54 ± 0.04	0.69 ± 0.06 *	29	0.25 ± 0.02	0.36 ± 0.03	0.46 ± 0.03 **	28
6G-FFT	7.41 ± 0.51	10.85 ± 0.98	8.66 ± 0.21 *	-20	4.16 ± 0.46	7.51 ± 0.96	9.00 ± 0.36	20	2.62 ± 0.19	3.14 ± 0.08	3.60 ± 0.02 ***	15
1-FFT	2.78 + 0.37	3.42 + 0.25	3.13 + 0.13 **	-22	0.52 + 0.07	1.05 + 0.06	1.23 + 0.10	17	0.74 + 0.03	0.71 + 0.04	0.96 + 0.01 ***	34
1-FEH	0.22 ± 0.01	0.18 + 0.01	0.15 + 0 00 ***	-18	0.15 ± 0.01	0.15 ± 0.00	0.18 ± 0.00 ***	23	0.13 ± 0.01	0.10 ± 0.01	0.13 + 0.03	28

Supplemental Table 4. Cell wall bound invertase (cwInv), vacuolar invertase (vacInv), cytoplasmic invertase (cytInv), phosphoglucose isomerase (PGI), phosphoglucose mutase (PGM), UDP-glucose pyrophosphorylase (UGPase), glucose 6-phosphat dehydrogenase (G6PDH), 1-sucrose:sucrose fructosyltransferase (1-SST), fructan:fructan 6G-fructosyltransferase (6G-FFT), fructan:fructan 1-fructosylstransferase (1-FFT) and fructan-1-exohydrolase (1-FEH) activities [nkat.gFW^{-1}] in stubble of 4-wk-old *Lolium perenne* Aberchoice plants harvested prior to treatment (Control), and 24 (24 hpt) or 48 hours (48 hpt) post treatment with water (Mock) or 10 µM kinetin. Enzymatic activities were analyzed in the first centimeter of stubble from ground level. Values in experiment 2 represent the mean of two, in experiments 1 and 3 the mean of three biological samples ± SE. Asterisks indicate values of enzymatic activities statistically different from mock treatment (analyzed by Student's t-test). * P < 0.05, ** P < 0.01, *** P < 0.001. Percent change in enzymatic activities between mock and kinetin treatment is given as Δ [%]. NA = no activity detected.

Enzyme	Specific activity [nkat.gFW^{-1}] Experiment 1			Δ [%]	Specific activity [nkat.gFW^{-1}] Experiment 2			Δ [%]	Specific activity [nkat.gFW^{-1}] Experiment 3			Δ [%]
	Control	Mock	Kinetin		Control	Mock	Kinetin		Control	Mock	Kinetin	
	0 hpt	24 hpt	24 hpt		0 hpt	24 hpt	24 hpt		0 hpt	24 hpt	24 hpt	
cwInv	0.46 ± 0.04	0.65 ± 0.13	0.53 ± 0.11	-15	0.69 ± 0.07	0.51 ± 0.07	0.57 ± 0.18	10	0.74 ± 0.18	0.89 ± 0.16	0.84 ± 0.04	-10
vacInv	0.88 ± 0.04	0.63 ± 0.07	1.18 ± 0.05 ***	74	1.31 ± 0.11	1.29 ± 0.06	1.19 ± 0.05	-8	0.81 ± 0.04	0.67 ± 0.04	0.53 ± 0.04 **	-20
cytInv	0.55 ± 0.03	0.51 ± 0.07	0.82 ± 0.07 ***	63	0.36 ± 0.03	0.39 ± 0.03	0.33 ± 0.01	-16	0.23 ± 0.03	0.19 ± 0.04	0.09 ± 0.03 ***	-54
PGI	8.06 ± 0.19	7.89 ± 0.44	7.24 ± 0.14	-8	18.62 ± 0.50	13.43 ± 0.37	11.44 ± 0.45 **	15	11.91 ± 0.31	11.41 ± 0.31	10.96 ± 025	-4
PGM	20.35 ± 0.21	17.20 ± 1.78	21.86 ± 0.68 *	27	21.86 ± 0.61	20.22 ± 0.71	21.43 ± 0.44	6	36.29 ± 0.52	26.36 + 1.61	32.05 ± 1.03 **	22
UGPase	37.31 ± 0.97	37.81 ± 1.75	33.22 ± 2.60	-12	51.90 ± 5.49	25.24 ± 1.93	33.26 ± 7.54	32	27.15 ± 1.38	33.51 + 4.38	38.18 ± 6.16	14
G6PDH	3.28 ± 0.19	2.27 ± 0.33	3.41 ± 0.23 **	50	4.42 ± 0.28	2.48 ± 0.19	2.65 ± 0.16	7	NA	NA	NA	NA
1-SST	0.25 ± 0.02	0.22 ± 0.01	0.76 ± 0.04 ***	248	0.74 ± 0.06	0.67 ± 0.03	1.02 ± 0.04 *	53	0.59 ± 0.03	0.68 + 0.02	0.54 ± 0.03 **	-21
6G-FFT	2.62 ± 0.10	2.79 ± 0.17	4.36 ± 0.13 ***	56	5.51 ± 0.26	4.03 ± 0.16	8.08 ± 0.16 **	100	6.11 ± 0.18	5.91 + 0.13	5.25 ± 0.15 **	-11
1-FFT	0.74 ± 0.03	0.81 ± 0.06	1.28 ± 0.06 ***	59	1.40 ± 0.12	0.73 ± 0.04	2.08 ± 0.08 ***	230	1.22 ± 0.06	1.16 + 0.05	0.94 ± 0.06 **	-19
1-FEH	0.13 ± 0.01	0.12 ± 0.01	0.17 ± 0.01 ***	39	0.11 ± 0.00	0.10 ± 0.00	0.12 ± 0.01 **	17	NA	NA	NA	NA
	0 hpt	48 hpt	48 hpt		0 hpt	48 hpt	48 hpt		0 hpt	48 hpt	48 hpt	
cwInv	0.46 ± 0.02	0.88 ± 0.06	0.98 ± 0.02	11	0.69 ± 0.07	0.44 ± 0.02	0.81 ± 0.04 ***	85	0.74 ± 0.09	0.69 ± 0.06	0.97 ± 0.07 **	40
vacInv	0.88 ± 0.04	0.66 ± 0.04	0.98 ± 0.02 ***	49	1.31 ± 0.11	1.05 ± 0.08	0.81 ± 0.04 **	-22	0.81 ± 0.04	0.67 ± 0.04	0.97 ± 0.06 ***	46
cytInv	0.55 ± 0.03	0.33 ± 0.04	0.56 ± 0.04 ***	68	0.36 ± 0.03	0.27 ± 0.02	0.25 ± 0.02	-8	0.23 ± 0.03	0.19 ± 0.04	0.19 ± 0.03	0
PGI	8.06 ± 0.19	7.32 ± 0.22	6.90 ± 0.25	-6	18.62 ± 0.50	11.28 ± 0.40	11.68 ± 0.56	3	11.91 ± 0.31	9.81 ± 0.34	7.31 ± 32.46	-25
PGM	20.35 ± 0.21	18.35 ± 0.21	19.66 ± 0.35 ***	-11	21.86 ± 0.61	20.22 ± 0.62	20.61 ± 0.43 **	12	36.29 ± 0.52	26.36 + 1.61	25.85 ± 0.94	-2
UGPase	37.31 ± 0.97	54.96 ± 2.08	49.38 ± 2.28	-10	51.90 ± 5.49	40.50 ± 8.08	37.47 ± 7.13	-7	27.15 ± 1.38	33.51 ± 4.38	23.30 ± 2.44 *	-30
G6PDH	3.28 ± 0.19	2.95 ± 0.22	2.53 ± 0.14	-14	4.42 ± 0.28	3.44 ± 0.26	2.90 ± 0.32	-16	NA	NA	NA	NA
1-SST	0.25 ± 0.02	0.36 ± 0.03	0.32 ± 0.01	-12	0.74 ± 0.06	0.69 ± 0.00	0.88 ± 0.02	28	0.59 ± 0.03	0.68 ± 0.02	0.81 ± 0.05 *	18
6G-FFT	2.62 ± 0.10	3.14 ± 0.08	3.60 ± 0.04 ***	15	5.51 ± 0.26	6.15 ± 0.31	7.50 ± 0.16	22	6.11 ± 0.18	5.91 ± 0.13	6.64 ± 0.50	12
1-FFT	0.74 ± 0.03	0.71 ± 0.04	0.99 ± 0.01 ***	39	1.40 ± 0.12	1.48 ± 0.20	1.23 ± 0.06	-17	1.22 ± 0.06	1.16 + 0.05	1.36 ± 0.22	18
1-FEH	0.13 ± 0.01	0.10 ± 0.01	0.13 ± 0.01	29	0.11 ± 0.00	0.11 ± 0.01	0.13 ± 0.01	23	NA	NA	NA	NA

Supplemental Table 5. Cell wall bound invertase (cwInv), vacuolar invertase (vacInv), cytoplasmic invertase (cytInv), phosphoglucose isomerase (PGI), phosphoglucose mutase (PGM), UDP-glucose pyrophosphorylase (UGPase), glucose 6-phosphat dehydrogenase (G6PDH), 1-sucrose:sucrose fructosyltransferase (1-SST), fructan:fructan 6G-fructosyltransferase (6G-FFT), fructan:fructan 1-fructosylstransferase (1-FFT) and fructan-1-exohydrolase (1-FEH) activities [nkat.gFW^{-1}] in stubble of 4-wk-old *Lolium perenne* Aberchoice plants harvested prior to treatment (Control), and 24 (24 hpt) or 48 hours (48 hpt) post treatment with water (Mock) or 10 µM gibberellic acid. Enzymatic activities were analyzed in the first centimeter of stubble from ground level. Values in experiment 1 and experiment 2 48 hpt represent the mean of two, in experiments 2 and 3 the mean of three biological samples ± SE. Asterisks indicate values of enzymatic activities statistically different from mock treatment (analyzed by Student's t-test). * P < 0.05, ** P < 0.01, *** P < 0.001. Percent change in enzymatic activities between mock and gibberellic acid treatment is given as Δ [%].

Enzyme	Specific activity [nkat.gFW^{-1}] Experiment 1			Δ [%]	Specific activity [nkat.gFW^{-1}] Experiment 2			Δ [%]	Specific activity [nkat.gFW^{-1}] Experiment 3			Δ [%]
	Control	Mock	Gibberellic acid		Control	Mock	Gibberellic acid		Control	Mock	Gibberellic acid	
	0 hpt	24 hpt	24 hpt		0 hpt	24 hpt	24 hpt		0 hpt	24 hpt	24 hpt	
cwInv	0.61 ± 0.07	0.36 ± 0.02	0.52 ± 0.01 ***	46	1.10 ± 0.11	0.80 ± 0.11	0.43 ± 0.03 **	-46	0.69 ± 0.07	0.51 ± 0.03	0.49 ± 0.05	-5
vacInv	0.84 ± 0.11	0.62 ± 0.02	0.86 ± 0.06 **	38	0.59 ± 0.03	0.49 ± 0.02	0.73 ± 0.01 ***	48	1.31 ± 0.11	1.29 ± 0.06	1.21 ± 0.06	-7
cytInv	0.30 ± 0.01	0.20 ± 0.04	0.39 ± 0.02 **	89	0.28 ± 0.01	0.21 ± 0.02	0.28 ± 0.02 **	31	0.36 ± 0.03	0.39 ± 0.03	0.37 ± 0.02	-6
PGI	23.64 ± 2.74	24.28 ± 0.37	23.36 ± 1.23	-4	15.44 ± 0.44	18.69 ± 0.43	14.45 ± 0.42 ***	-23	18.62 ± 0.50	13.43 ± 0.37	15.28 ± 0.19 ***	14
PGM	45.81 ± 3.64	37.75 ± 0.73	45.20 ± 3.46	20	39.37 ± 1.39	40.85 ± 0.67	34.29 ± 1.14 ***	-16	21.86 ± 0.61	20.22 ± 0.71	22.02 ± 0.23 *	9
UGPase	65.78 ± 12.06	68.32 ± 1.17	89.76 ± 3.16 **	31	63.17 ± 5.90	66.04 ± 10.85	48.96 ± 5.23	-26	51.90 ± 5.49	25.24 ± 1.93	41.04 ± 6.17 *	63
G6PDH	2.96 ± 0.84	5.30 ± 0.54	7.17 ± 0.06 **	35	5.19 ± 0.23	5.15 ± 0.22	4.45 ± 0.12 **	-14	4.42 ± 0.28	2.48 ± 0.19	3.60 ± 0.38 *	45
1-SST	1.51 ± 0.03	2.04 ± 0.08	2.89 ± 0.41 *	42	0.46 ± 0.07	0.41 ± 0.01	0.72 ± 0.02 ***	74	0.74 ± 0.06	0.67 ± 0.03	0.57 ± 0.04	-14
6G-FFT	7.41 ± 0.36	7.35 ± 0.62	8.32 ± 0.78	13	4.16 ± 0.23	5.01 ± 0.15	6.98 ± 0.08 ***	39	5.51 ± 0.26	4.03 ± 0.16	4.38 ± 0.04 *	9
1-FFT	2.78 ± 0.37	2.45 ± 0.08	2.98 ± 0.31	22	0.52 ± 0.07	0.81 ± 0.03	1.01 ± 0.04 ***	25	1.40 ± 0.12	0.73 ± 0.04	1.08 ± 0.07 ***	49
1-FEH	0.22 ± 0.01	0.20 ± 0.01	0.19 ± 0.02	-5	0.15 ± 0.01	0.14 ± 0.01	0.13 ± 0.01	-4	0.11 ± 0.00	0.10 ± 0.00	0.09 ± 0.00	-6
	0 hpt	48 hpt	48 hpt		0 hpt	48 hpt	48 hpt		0 hpt	48 hpt	48 hpt	
cwInv	0.61 ± 0.07	0.53 ± 0.01	0.43 ± 0.03 *	-18	1.10 ± 0.11	0.53 ± 0.11	0.40 ± 0.02	-24	0.69 ± 0.07	0.44 ± 0.02	1.36 ± 0.06 ***	209
vacInv	0.84 ± 0.11	0.63 ± 0.02	0.83 ± 0.11	32	0.59 ± 0.03	0.48 ± 0.02	0.63 ± 0.01 ***	30	1.31 ± 0.11	1.05 ± 0.08	1.36 ± 0.06 **	30
cytInv	0.30 ± 0.01	0.23 ± 0.02	0.33 ± 0.09	40	0.28 ± 0.01	0.46 ± 0.03	0.52 ± 0.04	13	0.36 ± 0.03	0.27 ± 0.02	0.40 ± 0.02 ***	49
PGI	23.64 ± 2.74	25.39 ± 0.62	23.67 ± 1.40	7	15.44 ± 0.44	21.48 ± 0.42	19.48 ± 0.81*	-9	18.62 ± 0.50	11.28 ± 0.40	11.85 ± 0.23	5
PGM	45.81 ± 3.64	47.78 ± 3.04	46.89 ± 3.99	-2	39.37 ± 1.39	45.74 ± 0.77	36.36 ± 1.58 ***	-21	21.86 ± 0.61	20.22 ± 0.62	19.66 ± 0.33	7
UGPase	65.78 ± 12.06	80.45 ± 0.64	91.68 ± 3.55	14	63.17 ± 5.90	55.42 ± 4.05	75.60 ± 3.80 ***	36	51.90 ± 5.49	40.50 ± 8.08	54.21 ± 4.39	34
G6PDH	2.96 ± 0.84	5.79 ± 0.55	7.23 ± 0.06	25	5.19 ± 0.23	4.51 ± 0.17	5.21 ± 0.27 *	16	4.42 ± 0.28	3.44 ± 0.26	3.25 ± 0.16	-6
1-SST	1.51 ± 0.03	2.47 ± 0.22	1.56 ± 0.04 **	-37	0.46 ± 0.07	0.54 ± 0.04	0.52 ± 0.08	-4	0.74 ± 0.06	0.69 ± 0.00	0.97 ± 0.12 *	40
6G-FFT	7.41 ± 0.36	10.85 ± 0.98	7.14 ± 0.77 **	-34	4.16 ± 0.23	7.51 ± 0.96	7.73 ± 0.11	3	5.51 ± 0.26	6.15 ± 0.31	8.35 ± 1.04	36
1-FFT	2.78 ± 0.37	3.42 ± 0.25	2.07 ± 0.04 ***	-39	0.52 ± 0.07	1.05 ± 0.06	1.21 ± 0.04	15	1.40 ± 0.12	1.48 ± 0.20	1.87 ± 0.29	26
1-FEH	0.22 ± 0.01	0.18 ± 0.01	0.12 ± 0.01 **	-34	0.15 ± 0.01	0.15 ± 0.00	0.17 ± 0.01 *	19	0.11 ± 0.00	0.11 ± 0.01	0.07 ± 0.01 **	-35

Supplemental Table 6. Carbohydrate concentrations [mg.gFW^{-1}] in stubble of 4-wk-old *Lolium perenne* Aberchoice plants harvested prior to treatment (Control), and 24 (24 hpt) or 48 hours (48 hpt) post treatment with water (Mock) or 10 µM abscisic acid. Carbohydrate concentrations were analyzed in the first centimeter of stubble from ground level. Values represent the mean of three biological samples ± SE. Asterisks indicate values of carbohydrate concentrations statistically different from mock treatment (analyzed by Student's t-test). * $P < 0.05$, ** $P < 0.01$, *** $P < 0.001$. Percent change in carbohydrate concentrations between mock and abscisic acid treatment is given as Δ [%].

Carbohydrate	Concentration [mg.gFW^{-1}] Experiment 1			Δ [%]	Concentration [mg.gFW^{-1}] Experiment 2			Δ [%]	Concentration [mg.gFW^{-1}] Experiment 3			Δ [%]
	Control	Mock	Abscisic acid		Control	Mock	Abscisic acid		Control	Mock	Abscisic acid	
	0 hpt	24 hpt	24 hpt		0 hpt	24 hpt	24 hpt		0 hpt	24 hpt	24 hpt	
Glucose	0.63 ± 0.05	0.46 ± 0.05	0.64 ± 0.10	41	1.97 ± 0.10	1.53 ± 0.07	1.60 ± 0.15	5	1.14 ± 0.06	1.02 ± 0.06	1.38 ± 0.09	36
Fructose	0.21 ± 0.05	0.20 ± 0.04	0.23 ± 0.07	17	1.71 ± 0.09	1.12 ± 0.21	1.29 ± 0.17	15	0.90 ± 0.10	0.76 ± 0.14	0.82 ± 0.08	8
Sucrose	2.40 ± 0.39	2.06 ± 0.20	2.34 ± 0.75	14	2.98 ± 0.29	3.19 ± 0.41	2.63 ± 0.40	-18	5.14 ± 0.41	4.73 ± 0.50	4.79 ± 0.49	1
Raffinose	0.09 ± 0.01	0.07 ± 0.01	0.09 ± 0.01	26	0.32 ± 0.01	0.25 ± 0.01	0.22 ± 0.02	-9	0.17 ± 0.02	0.15 ± 0.01	0.16 ± 0.01	5
Low DP fructans	1.38 ± 0.04	1.11 ± 0.08	1.39 ± 0.09 *	25	1.28 ± 0.08	1.35 ± 0.02	1.39 ± 0.07	3	1.67 ± 0.03	1.61 ± 0.03	1.79 ± 0.19	12
	0 hpt	48 hpt	48 hpt		0 hpt	48 hpt	48 hpt		0 hpt	48 hpt	48 hpt	
Glucose	0.63 ± 0.05	0.73 ± 0.09	0.65 ± 0.06	-11	1.97 ± 0.10	2.06 ± 0.20	2.03 ± 0.21	-1	1.14 ± 0.06	1.24 ± 0.10	1.18 ± 0.13	-5
Fructose	0.21 ± 0.05	0.25 ± 0.08	0.21 ± 0.05	-15	1.71 ± 0.09	1.69 ± 0.09	1.64 ± 0.28	-3	0.90 ± 0.10	0.77 ± 0.10	0.83 ± 0.11	7
Sucrose	2.40 ± 0.39	2.73 ± 0.47	2.53 ± 0.44	-7	2.98 ± 0.29	3.53 ± 1.14	3.55 ± 0.22	0	5.14 ± 0.41	5.81 ± 0.60	5.10 ± 0.61	-12
Raffinose	0.09 ± 0.01	0.09 ± 0.01	0.10 ± 0.01	7	0.32 ± 0.01	0.26 ± 0.03	0.23 ± 0.01	-13	0.17 ± 0.02	0.19 ± 0.02	0.17 ± 0.02	-8
Low DP fructans	1.38 ± 0.04	1.86 ± 0.12	1.50 ± 0.10 *	-20	1.28 ± 0.08	1.64 ± 0.27	1.44 ± 0.09	-12	1.67 ± 0.03	1.93 ± 0.14	1.61 ± 0.02	-17

Supplemental Table 7. Carbohydrate concentrations [mg.gFW^{-1}] in stubble of 4-wk-old *Lolium perenne* Aberchoice plants harvested prior to treatment (Control), and 24 (24 hpt) or 48 hours (48 hpt) post treatment with water (Mock) or 0.15% ethephon. Carbohydrate concentrations were analyzed in the first centimeter of stubble from ground level. Values represent the mean of three biological samples ± SE. Asterisks indicate values of carbohydrate concentrations statistically different from mock treatment (analyzed by Student's t-test). * P < 0.05, ** P < 0.01, *** P < 0.001. Percent change in carbohydrate concentrations between mock and ethephon treatment is given as Δ [%].

Carbohydrate	Concentration [mg.gFW^{-1}]			Δ [%]	Concentration [mg.gFW^{-1}]			Δ [%]	Concentration [mg.gFW^{-1}]			Δ [%]
	Experiment 1				Experiment 2				Experiment 3			
	Control	Mock	Ethephon		Control	Mock	Ethephon		Control	Mock	Ethephon	
	0 hpt	24 hpt	24 hpt		0 hpt	24 hpt	24 hpt		0 hpt	24 hpt	24 hpt	
Glucose	0.63 ± 0.05	0.46 ± 0.05	0.58 ± 0.06	27	1.97 ± 0.10	1.53 ± 0.07	2.04 ± 0.08 *	33	1.14 ± 0.06	1.02 ± 0.06	1.20 ± 0.09	18
Fructose	0.21 ± 0.05	0.20 ± 0.04	0.19 ± 0.04	-4	1.71 ± 0.09	1.12 ± 0.21	1.81 ± 0.20 *	62	0.90 ± 0.10	0.76 ± 0.14	0.76 ± 0.08	0
Sucrose	2.40 ± 0.39	2.06 ± 0.20	2.70 ± 0.27	31	2.98 ± 0.29	3.19 ± 0.41	2.86 ± 0.32	-10	5.14 ± 0.41	4.73 ± 0.50	4.91 ± 0.28	4
Raffinose	0.09 ± 0.01	0.07 ± 0.01	0.09 ± 0.01	22	0.32 ± 0.01	0.25 ± 0.01	0.26 ± 0.01	7	0.17 ± 0.02	0.15 ± 0.01	0.17 ± 0.01	11
Low DP fructans	1.38 ± 0.04	1.11 ± 0.08	1.45 ± 0.08 *	30	1.28 ± 0.08	1.35 ± 0.02	1.44 ± 0.06	7	1.67 ± 0.03	1.61 ± 0.03	1.56 ± 0.06	-3
	0 hpt	48 hpt	48 hpt		0 hpt	48 hpt	48 hpt		0 hpt	48 hpt	48 hpt	
Glucose	0.63 ± 0.05	0.73 ± 0.09	0.66 ± 0.06	-10	1.97 ± 0.10	2.06 ± 0.20	2.10 ± 0.02	2	1.14 ± 0.06	1.24 ± 0.10	1.33 ± 0.16	7
Fructose	0.21 ± 0.05	0.25 ± 0.08	0.22 ± 0.05	-13	1.71 ± 0.09	1.12 ± 0.21	1.84 ± 0.16	9	0.90 ± 0.10	0.76 ± 0.14	0.84 ± 0.11	9
Sucrose	2.40 ± 0.39	2.73 ± 0.47	2.78 ± 0.39	2	2.98 ± 0.29	3.53 ± 1.14	3.83 ± 0.03	8	5.14 ± 0.41	5.81 ± 0.60	5.25 ± 0.56	-10
Raffinose	0.09 ± 0.01	0.09 ± 0.01	0.08 ± 0.01	-10	0.32 ± 0.01	0.26 ± 0.03	0.29 ± 0.01	9	0.17 ± 0.02	0.19 ± 0.02	0.17 ± 0.01	-10
Low DP fructans	1.38 ± 0.04	1.86 ± 0.12	1.59 ± 0.15	-15	1.28 ± 0.08	1.64 ± 0.27	1.53 ± 0.04	-7	1.67 ± 0.03	1.93 ± 0.14	1.62 ± 0.13	-16

Supplemental Table 8. Carbohydrate concentrations [mg.gFW^{-1}] in stubble of 4-wk-old *Lolium perenne* Aberchoice plants harvested prior to treatment (Control), and 24 (24 hpt) or 48 hours (48 hpt) post treatment with water (Mock) or 10 µM auxin. Carbohydrate concentrations were analyzed in the first centimeter of stubble from ground level. Values represent the mean of three biological samples ± SE. Asterisks indicate values of carbohydrate concentrations statistically different from mock treatment (analyzed by Student's t-test). * P < 0.05, ** P < 0.01, *** P < 0.001. Percent change in carbohydrate concentrations between mock and auxin treatment is given as Δ [%].

Carbohydrate	Concentration [mg.gFW^{-1}]			Δ [%]	Concentration [mg.gFW^{-1}]			Δ [%]	Concentration [mg.gFW^{-1}]			Δ [%]
	Experiment 1				Experiment 2				Experiment 3			
	Control	Mock	Auxin		Control	Mock	Auxin		Control	Mock	Auxin	
	0 hpt	24 hpt	24 hpt		0 hpt	24 hpt	24 hpt		0 hpt	24 hpt	24 hpt	
Glucose	0.63 ± 0.05	0.46 ± 0.05	0.49 ± 0.06	7	1.97 ± 0.10	1.53 ± 0.07	1.94 ± 0.08 *	27	1.14 ± 0.06	1.02 ± 0.06	1.13 ± 0.16	12
Fructose	0.21 ± 0.05	0.20 ± 0.04	0.16 ± 0.04	-17	1.71 ± 0.09	1.12 ± 0.21	1.50 ± 0.10	34	0.90 ± 0.10	0.76 ± 0.14	0.81 ± 0.14	6
Sucrose	2.40 ± 0.39	2.06 ± 0.20	1.94 ± 0.05	-6	2.98 ± 0.29	3.19 ± 0.41	2.43 ± 0.09	-24	5.14 ± 0.41	4.73 ± 0.50	4.40 ± 0.24	-7
Raffinose	0.09 ± 0.01	0.07 ± 0.01	0.10 ± 0.02	32	0.32 ± 0.01	0.25 ± 0.01	0.24 ± 0.01	-3	0.17 ± 0.02	0.15 ± 0.01	0.17 ± 0.00	8
Low DP fructans	1.38 ± 0.04	1.11 ± 0.08	1.22 ± 0.07	10	1.28 ± 0.08	1.35 ± 0.02	1.40 ± 0.06	4	1.67 ± 0.03	1.61 ± 0.03	1.67 ± 0.02	4
	0 hpt	48 hpt	48 hpt		0 hpt	48 hpt	48 hpt		0 hpt	48 hpt	48 hpt	
Glucose	0.63 ± 0.05	0.73 ± 0.09	1.23 ± 0.41	67	1.97 ± 0.10	2.06 ± 0.20	2.14 ± 0.11	4	1.14 ± 0.06	1.24 ± 0.10	1.25 ± 0.19	1
Fructose	0.21 ± 0.05	0.25 ± 0.08	0.28 ± 0.10	13	1.71 ± 0.09	1.12 ± 0.21	1.93 ± 0.14	14	0.90 ± 0.10	0.76 ± 0.14	0.94 ± 0.17	21
Sucrose	2.40 ± 0.39	2.73 ± 0.47	2.85 ± 0.23	4	2.98 ± 0.29	3.53 ± 1.14	2.71 ± 0.30	-23	5.14 ± 0.41	5.81 ± 0.60	5.06 ± 0.61	-13
Raffinose	0.09 ± 0.01	0.09 ± 0.01	0.12 ± 0.04	31	0.32 ± 0.01	0.26 ± 0.03	0.22 ± 0.01	-16	0.17 ± 0.02	0.19 ± 0.02	0.18 ± 0.01	-3
Low DP fructans	1.38 ± 0.04	1.86 ± 0.12	1.96 ± 0.20	5	1.28 ± 0.08	1.64 ± 0.27	1.37 ± 0.06	-16	1.67 ± 0.03	1.93 ± 0.14	1.75 ± 0.02	-9

Supplemental Table 9. Carbohydrate concentrations [mg.gFW^{-1}] in stubble of 4-wk-old *Lolium perenne* Aberchoice plants harvested prior to treatment (Control), and 24 (24 hpt) or 48 hours (48 hpt) post treatment with water (Mock) or 10 µM kinetin. Carbohydrate concentrations were analyzed in the first centimeter of stubble from ground level. Values in experiment one 24 hpt represent the mean of two biological samples, values at 48 hpt represent one biological sample (ND = not enough data for statistical analysis), values in experiments two and three represent the mean of three biological samples ± SE. Asterisks indicate values of carbohydrate concentrations statistically different from mock treatment (analyzed by Student's t-test). * P < 0.05, ** P < 0.01, *** P < 0.001. Percent change in carbohydrate concentrations between mock and kinetin treatment is given as Δ [%].

Carbohydrate	Concentration [mg.gFW^{-1}]			Δ [%]	Concentration [mg.gFW^{-1}]			Δ [%]	Concentration [mg.gFW^{-1}]			Δ [%]
	Experiment 1				Experiment 2				Experiment 3			
	Control	Mock	Kinetin		Control	Mock	Kinetin		Control	Mock	Kinetin	
	0 hpt	24 hpt	24 hpt		0 hpt	24 hpt	24 hpt		0 hpt	24 hpt	24 hpt	
Glucose	0.52 ± 0.04	0.39 ± 0.04	0.26 ± 0.19	-32	0.85 ± 0.03	0.79 ± 0.04	0.73 ± 0.03 *	-8	1.14 ± 0.06	1.02 ± 0.06	1.89 ± 0.09 *	86
Fructose	0.34 ± 0.01	0.26 ± 0.00	0.18 ± 0.14	-29	0.32 ± 0.01	0.29 ± 0.02	0.34 ± 0.01	16	0.90 ± 0.10	0.76 ± 0.14	1.16 ± 0.05	53
Sucrose	3.61 ± 0.16	2.94 ± 017	2.04 ± 0.85	-31	4.05 ± 0.28	4.08 ± 0.20	4.76 ± 0.85	16	5.14 ± 0.41	4.73 ± 0.50	5.81 ± 0.14	23
Raffinose	0.09 ± 0.01	0.09 ± 0.01	0.08 ± 0.04	-14	0.08 ± 0.01	0.06 ± 0.00	0.07 ± 0.01	15	0.17 ± 0.02	0.15 ± 0.01	0.19 ± 0.01 *	23
Low DP fructans	0.98 ± 0.10	1.39 ± 0.04	0.81 ± 0.33	-21	1.40 ± 0.08	1.39 ± 0.04	1.15 ± 0.03	-17	1.67 ± 0.03	1.61 ± 0.03	2.05 ± 0.04 *	27
	0 hpt	48 hpt	48 hpt		0 hpt	48 hpt	48 hpt		0 hpt	48 hpt	48 hpt	
Glucose	0.52 ± 0.04	0.56 ± 0.05	0.51 ND	-9	0.85 ± 0.03	0.79 ± 0.04	0.65 ± 0.03 *	-18	1.14 ± 0.06	1.24 ± 0.15	1.10 ± 0.10	-11
Fructose	0.34 ± 0.01	0.32 ± 0.02	0.31 ND	-3	0.32 ± 0.01	0.29 ± 0.02	0.29 ± 0.02	0	0.90 ± 0.10	0.77 ± 0.10	0.94 ± 0.17	21
Sucrose	3.61 ± 0.16	3.43 ± 0.06	2.96 ND	-4	4.05 ± 0.28	4.08 ± 0.20	4.04 ± 0.04	-1	5.14 ± 0.41	5.81 ± 0.60	4.29 ± 0.37	-26
Raffinose	0.09 ± 0.01	0.09 ± 0.00	0.07 ND	-17	0.08 ± 0.01	0.06 ± 0.00	0.08 ± 0.01	25	0.17 ± 0.02	0.19 ± 0.02	0.16 ± 0.01	-11
Low DP fructans	0.98 ± 0.10	1.27 ± 0.33	0.49 ND	-62	1.40 ± 0.08	1.39 ± 0.04	1.34 ± 0.00	-3	1.67 ± 0.03	1.93 ± 0.14	1.64 ± 0.09	-15

Supplemental Table 10. Carbohydrate concentrations [mg.gFW^{-1}] in stubble of 4-wk-old *Lolium perenne* Aberchoice plants harvested prior to treatment (Control), and 24 (24 hpt) or 48 hours (48 hpt) post treatment with water (Mock) or 10 µM gibberellic acid. Carbohydrate concentrations were analyzed in the first centimeter of stubble from ground level. Values in experiment three 24 hpt represent the mean of two biological samples, values in experiments one and three represent the mean of three biological samples ± SE. Values at 48 hpt in experiment three represent one biological sample (ND = not enough data for statistical analysis). Asterisks indicate values of carbohydrate concentrations statistically different from mock treatment (analyzed by Student's t-test). * P < 0.05, ** P < 0.01, *** P < 0.001. Percent change in carbohydrate concentrations between mock and gibberellic acid treatment is given as Δ [%].

Carbohydrate	Concentration [mg.gFW^{-1}]			Δ [%]	Concentration [mg.gFW^{-1}]			Δ [%]	Concentration [mg.gFW^{-1}]			Δ [%]
	Experiment 1				Experiment 2				Experiment 3			
	Control	Mock	Gibberellic acid		Control	Mock	Gibberellic acid		Control	Mock	Gibberellic acid	
	0 hpt	24 hpt	24 hpt		0 hpt	24 hpt	24 hpt		0 hpt	24 hpt	24 hpt	
Glucose	0.63 ± 0.05	0.46 ± 0.05	0.65 ± 0.03	43	1.97 ± 0.10	1.53 ± 0.07	2.12 ± 0.01 **	39	0.52 ± 0.04	0.39 ± 0.04	0.52 ± 0.03	33
Fructose	0.21 ± 0.05	0.20 ± 0.04	0.19 ± 0.03	-4	1.71 ± 0.09	1.12 ± 0.21	1.59 ± 0.14	42	0.34 ± 0.01	0.26 ± 0.00	0.36 ± 0.05	39
Sucrose	2.40 ± 0.39	2.06 ± 0.20	2.07 ± 0.16	1	2.98 ± 0.29	3.19 ± 0.41	2.50 ± 0.33	-22	3.61 ± 0.16	2.94 ± 017	3.43 ± 0.05	16
Raffinose	0.09 ± 0.01	0.07 ± 0.01	0.05 ± 0.00	-31	0.32 ± 0.01	0.25 ± 0.01	0.23 ± 0.03	-5	0.09 ± 0.01	0.09 ± 0.01	0.09 ± 0.01	0
Low DP fructans	1.38 ± 0.04	1.11 ± 0.08	0.81 ± 0.33	-21	1.28 ± 0.08	1.35 ± 0.02	1.28 ± 0.08	-17	0.98 ± 0.10	1.02 ± 0.09	1.18 ± 0.08	16
	0 hpt	48 hpt	48 hpt		0 hpt	48 hpt	0 hpt					
Glucose	0.63 ± 0.05	0.73 ± 0.09	0.82 ± 0.03	12	1.97 ± 0.10	2.06 ± 0.20	1.98 ± 0.17	-4	ND	ND	ND	
Fructose	0.21 ± 0.05	0.25 ± 0.08	0.28 ± 0.04	14	1.71 ± 0.09	1.69 ± 0.09	1.87 ± 0.03	11	ND	ND	ND	
Sucrose	2.40 ± 0.39	2.73 ± 0.47	2.73 ± 0.22	0	2.98 ± 0.29	3.53 ± 1.14	2.88 ± 0.20	-18	ND	ND	ND	
Raffinose	0.09 ± 0.01	0.09 ± 0.01	0.13 ± 0.04	44	0.32 ± 0.01	0.26 ± 0.03	0.23 ± 0.01	-14	ND	ND	ND	
Low DP fructans	1.38 ± 0.04	1.86 ± 0.12	1.97 ± 0.26	6	1.28 ± 0.08	1.64 ± 0.27	1.61 ± 0.01	-2	ND	ND	ND	
